# Targeting hormone treatment for the brain in menopause

**DOI:** 10.1038/s41598-026-65603-4

**Published:** 2026-09-01

**Authors:** Noriko Itoh, Yuichiro Itoh, Rajita Patil, Christi Till, Marissa Smith, Jaclyn Schwartz, Brittany Davis, Erica Oberman, Brianne D. Romeroso, Rhonda R. Voskuhl

**Affiliations:** 1 Department of Neurology, David Geffen School of Medicine at UCLA, Gordon Neuroscience Research Building, Room 475D,635 Charles Young Drive South, Los Angeles, CA 90095-7334 USA; 2 Department of Obstetrics and Gynecology, David Geffen School of Medicine at UCLA, Los Angeles, CA USA; 3 The Cleopatrarx Foundation, Tulsa, Oklahoma USA

**Keywords:** Menopause Hormone Therapy (MRT), Menopause, Estriol, Estrogen receptor β, Cognition, Astrocyte, Neurology, Neuroscience

## Abstract

Estrogens are neuroprotective, and menopause may contribute to neurodegeneration. Estriol preferentially binds estrogen receptor beta (ERβ) in brain to induce neuroprotection. Previously, oral estriol improved cognitive processing speed and reduced cerebral cortex atrophy at treatment month twelve in women with multiple sclerosis (MS). Here, treatment with a blisterpack designed for healthy menopausal women which contains estriol and progesterone (Pearlpak) was assessed for its effect on cognitive domain-specific symptoms in a case series of twenty menopausal women (mean age 53.5 years). At treatment month twelve, there were significant improvements in self-reported brain fog, concentration, working memory, processing speed, verbal memory, and problem solving. A preclinical model explored mechanisms. Midlife female mice (age 12-13 months), previously ovariectomized either at 2 months or at 11 months of age, exhibited cognitive deficits on Morris Water Maze, with glial activation and synaptic loss on dorsal hippocampal pathology. Astrocytes had increased levels of Enolase 1 (ENO1), a key protein in glucose utilization. Increased ENO1 correlated with worse cognitive performance. Deletion of ERβ in astrocytes in gonadally intact midlife females recapitulated deleterious effects of ovariectomy. Conversely, treatment of midlife female mice with estriol from age 11 months to age 12 months reduced ENO1 expression in hippocampal astrocytes, reduced glial activation and synaptic loss, and improved cognitive performance. This preliminary study suggests that Pearlpak treatment for cognitive symptoms of menopause warrants further investigation given the safe use of these hormones in Europe and Asia for decades to reduce other menopausal symptoms.

**Supplementary Information:**

The online version contains supplementary material available at https://doi.org/10.1038/s41598-026-65603-4.

## Introduction

“Brain fog” occurs in 62% of menopausal women^1,2^. This entails cognitive domain-specific subjective symptoms as well as changes on objective cognitive testing by neuropsychologists. Relative worsening occurs when testing verbal memory, working memory, concentration, and processing speed, where scores are lower, but still within the normative range established for age, sex, and level of education^1,3,4^. There are not significant deficits in global cognitive function using assessments to diagnose dementia. That said, this cognitive domain-specific relative worsening undermines confidence in these women at work and at home.

While hot flashes, night sweats, and poor sleep are not good for cognition in the short term^5^, this is not mutually exclusive of a deleterious effect of menopause-induced loss of neuroprotective estrogen in the long term. With mean age of menopause onset of 51.5 years, women will be estrogen deficient for a third of their life (into their 80s). Standard menopause hormone therapy (MHT) using transdermal estradiol (patch) or oral conjugated equine estrogens (Premarin) did not show benefit on cognition, even when given in early menopause for four years duration in the Cognitive and Affective Study of the Kronos Early Estrogen Prevention Study (KEEPS-Cog), ancillary to the Women’s Health Initiative (WHI) study^6,7^. Together, cognitive issues of menopause are a concern for most menopausal women, and there is no FDA approved treatment for this indication.

Beyond clinical symptoms, there are structural and functional abnormalities of the brain on neuroimaging. A brain region affected by menopause is the hippocampus, a region involved in learning and memory. There is hippocampal subregion atrophy as well as abnormalities in functional outcomes^8–14^. Conversely, regional brain protection has been reported in women with more years of estrogen exposure (more time between menarche and menopause) or more exposure to high levels of estrogen during pregnancy (multiparity)^15,16^. Brain region-specific changes, not global brain changes, are consistent with cognitive domain-specific symptoms.

In a previously published mouse model, aging to midlife (months 12-14) combined with complete estrogen deficiency for ten months caused neurodegeneration in females. This included dorsal hippocampal atrophy on MRI with neuropathology therein, namely astrocyte and microglia activation with synaptic loss^17^. Transcriptomics in astrocytes showed that the most differentially expressed gene pathways were for glucose utilization (glycolysis and gluconeogenesis)^17^. Discovery of abnormal glucose utilization in hippocampal astrocytes in this preclinical model suggested this as a cellular and molecular mechanism underlying abnormalities in glucose and energy metabolism observed by neuroimaging in women with menopause^9–11,18^.

Estrogens have long been known to have neuroprotective properties, with receptor alpha (ERα) and estrogen receptor beta (ERβ) expressed in hippocampus and cortex of mice and humans^19–27^. However, the optimal type and dose of estrogen to treat cognitive issues of menopause remains unknown. Estradiol is an FDA approved treatment for hot flashes, not cognitive issues, of menopause. Estradiol is used at the lowest dose and shortest duration possible given the controversial concerns about its role in breast cancer risk^28,29^. Estradiol binds to, and strongly stimulates, estrogen receptor alpha (ERα) in ER positive (ER+) breast cancers, and blocking ERα stimulation is the target of breast cancer treatments using Tamoxifen and aromatase inhibitors^30,31^.

Estriol has been safely used to treat vasomotor symptoms of menopause in Europe and Asia for over 40 years^32–40^. Estriol binding to ERα is weaker than estradiol binding to ERα^41–43^. Estriol is the estrogen unique to pregnancy, binding primarily to estrogen receptor beta (ERβ). Estriol was previously tested in women with multiple sclerosis (MS), since the latter half of pregnancy is protective^44–47^. Preclinical studies in the MS mouse model used a dose of estriol treatment to recapitulate a late pregnancy level in serum of nonpregnant mice^48^. Estriol treatment in clinical trials of women with MS used a dose recapitulating mid-pregnancy serum levels, namely oral estriol 8mg per day^49^, a dose previously well tolerated in menopausal women^50^.

Estriol mediated protection was observed through anti-inflammatory effects in the immune system and neuroprotective effects in the brain^48,51–55^. Mechanisms of neuroprotection in the MS model showed that estriol treatment reduced regional brain atrophy in hippocampus and cerebral cortex^56,57^. On neuropathology, estriol treatment increased synapses and decreased neuronal loss in gray matter, while inducing remyelination and decreasing axonal damage in white matter^48,52,56,57^. Selective deletion of the ERβ gene showed that neuroprotection was mediated by ERβ in both microglia and oligodendrocytes^58–60^. Neuroprotective effects in Phase 2 clinical trials of oral estriol treatment in MS women included a multicenter, double-blinded, randomized, placebo-controlled trial. At month 12 of estriol treatment, there was improved cognitive processing speed, reduced cerebral cortex atrophy, and lower levels of a blood biomarker of neuroinflammation and neurodegeneration, each as compared to placebo^49,61–66^. Estriol represents novel repurposing of a treatment from MS women to otherwise healthy menopausal women.

This study takes a key step in addressing the unmet need for discovery of a treatment targeting cognitive domain-specific symptoms and brain region-specific pathologies of menopause.

## Results

### Pearlpak treatment improves cognitive domain-specific symptoms in menopausal women

**Fig. 1:**
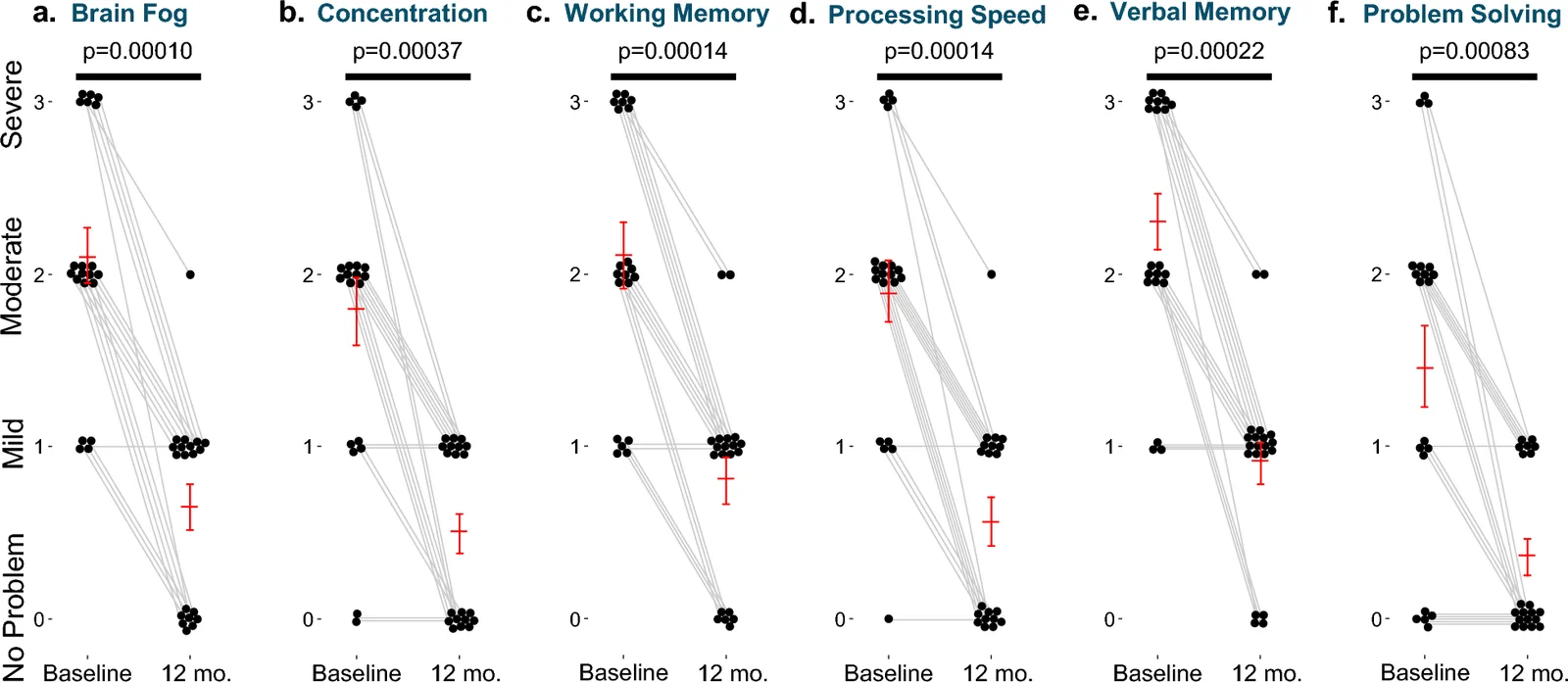
Treatment with Pearlpak in menopausal women. A questionnaire (Voskuhl Menopause Brain Fog Assessment) was administered to subjects to determine cognitive domain-specific symptoms in twenty menopausal women at two timepoints: before treatment (month 0) and during treatment (month 12). Grading was done for each cognitive symptom: (**a**) brain fog, (**b**) concentration, (**c**) working memory, (**d**) processing speed, (**e**) verbal memory, and (**f**) problem solving. Dots show each cognitive domain-specific score, with values before and during treatment from each woman, connected (paired) by a gray line to show change over time. Paired samples Wilcoxon signed rank test was used to determine significance of change in cognitive scores during treatment (12 months) compared with pretreatment baseline (month 0). Red lines indicate means and standard errors at each timepoint.

Twenty women, mean age was 53.5 years (SD = 8.6 years), were treated for twelve months with Pearlpak, blisterpacks designed for menopausal women containing estriol and progesterone (Table 1). All were treated with active drug (no placebo control, no dose randomization). Subjects were followed by the UCLA Comprehensive Menopause Program with in-person or telemedicine visits or by Cleopatrarx with telemedicine visits. The dose and timing of estriol and progesterone in blisterpacks was tailored for each subject with prioritization of treatment for cognition in context with the need for treatment of other menopausal symptoms (vasomotor symptoms, poor sleep, genitourinary syndrome) as well as age and other health issues. Estriol doses each morning were 2 mg (n = 10 subjects), 4 mg (n=7 subjects), or 8 mg (n=3 subjects). Progesterone (micronized) was taken at night at a dose of 100 mg (n=15 subjects), 200 mg (n=2 subjects) or none (no uterus or progesterone containing intrauterine device, n=3 subjects), for either 14 of each 28 day cycle or for all 28 days. Some individuals had tailored dose optimization adjustments during the first 3 months of starting treatment, with no further dose changes from month 4 to 12. Every subject completed the Voskuhl Menopause Brain Fog Assessment (Figure S1) at Baseline (month 0) and Follow-up (month 12). Subjects self-reported current cognitive domain-specific symptoms as either severe, moderate, mild, or no problem. In this case series, cognitive scores showed statistically significant improvement at treatment month 12 compared to pretreatment baseline month 0 in Brain Fog (r = 0.899, p = 0.00010, Fig. 1a), Concentration (r = 0.898, p = 0.00037, Fig. 1b), Working Memory (r = 0.905, p = 0.00014, Fig. 1c), Processing Speed (r = 0.903, p = 0.00014, Fig. 1d), Verbal Memory (r = 0.9, p = 0.00022, Fig. 1e), and Problem Solving (r = 0.903, p = 0.00083, Fig. 1f).

**Table 1:** Subject characteristics.

**Subject ID**	**Age**	**Education**	**Estriol**	**Progesterone**
	**(Year)**	**(16+ or 16 years)**	**Dose (mg)**	**Dose (mg)** **(14 or 28 days)**
**1**	45	16+	8	100 (28)
**2**	65	16	2	100 (14)
**3**	43	16	4	100 (14)
**4**	55	16	2	200 (28)
**5**	49	16	2	N/A
**6**	67	16	2	N/A
**7**	54	16+	2	100 (14)
**8**	61	16+	4	N/A
**9**	47	16	4	100 (28)
**10**	67	16	4	100 (28)
**11**	57	16+	8	100 (28)
**12**	50	16+	4	200 (28)
**13**	43	16	2	100 (28)
**14**	48	16+	8	100 (14)
**15**	60	16+	2	100 (28)
**16**	51	16	2	100 (28)
17^*^	36	16+	4	100 (28)
**18**	53	16	4	100 (28)
**19**	61	16	2	100 (14)
**20**	58	16	2	100 (14)

20 subjects, mean age 53.5 years (SD = 8.6 years), participated. Nineteen had natural menopause or perimenopause. One had surgical ovariectomy *. Education in years: 40% had 16+ years (beyond college degree); 60% had 16 years; 0% had less than 16 years. The estriol dose was 2 mg in ten subjects, 4 mg in seven subjects, and 8 mg in three subjects. The progesterone dose was 100 mg in fifteen subjects, and 200 mg in two subjects, and no progesterone in three subjects (N/A).

**Fig. 2:**
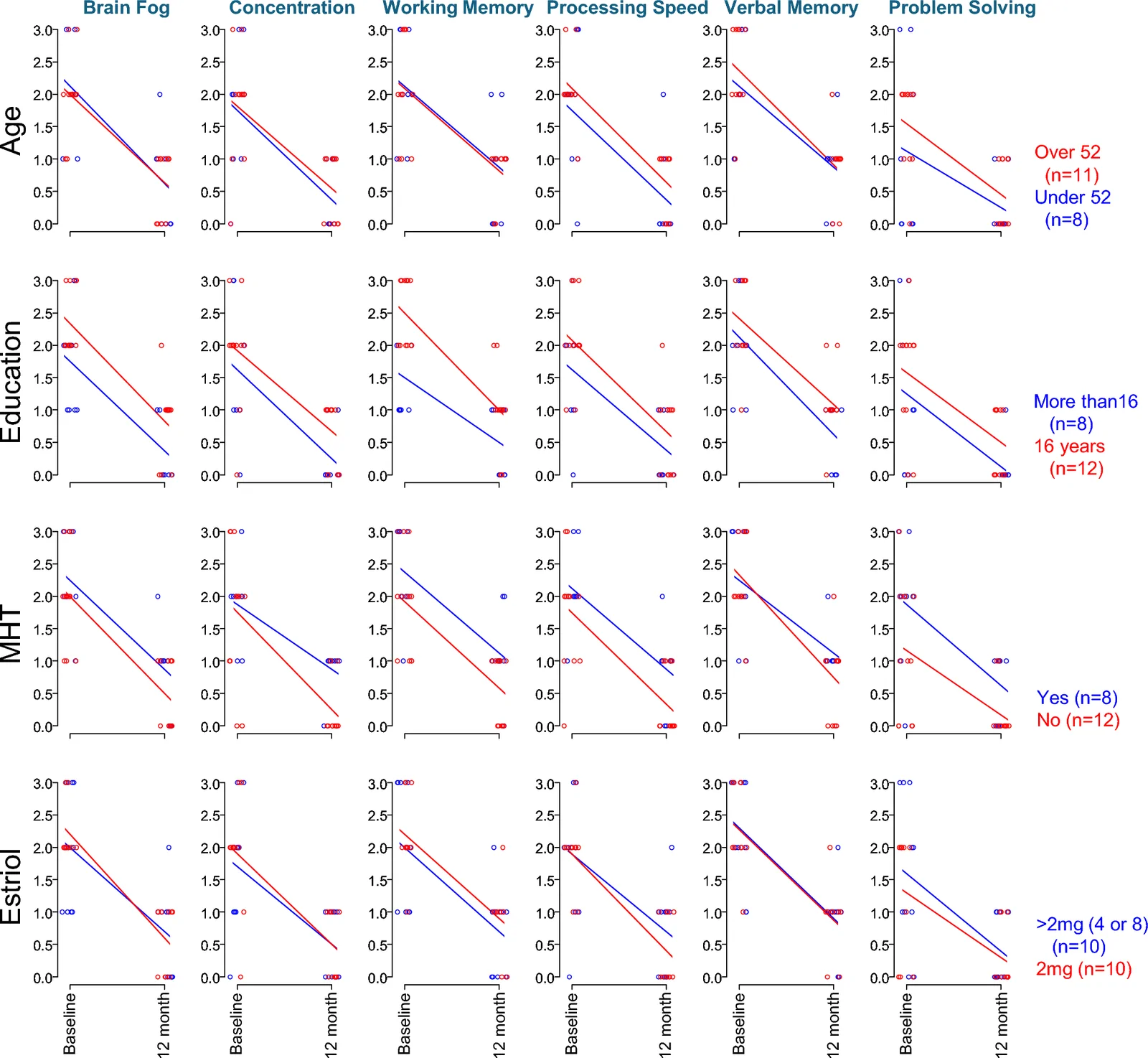
Cognitive domain-specific changes from pretreatment baseline month 0 to treatment month 12. Random effect components were prespecified as a set of covariates. Subjects were categorized by age (over 52 years versus under 52 years), education (more than 16 years versus 16 years), MHT using systemic estradiol treatment within the last 5 years (Yes versus No); or estriol dose (2mg, n=10 versus higher than 2 mg (4mg, n=7 or 8mg, n=3, total n=10). Regression lines for each subgroup are shown for each covariate. The only difference at baseline month 0 was the effect of education on working memory (p = 0.007, Welch’s Two-Sample t-test). All subgroups reported less severe symptoms at month 12 compared to month 0. There were no differences in improvement when comparing the two subgroups within each covariate (p > 0.05, Welch’s t-test on slopes from linear regression models). One subject (Table 1 indicated by *) was removed from the analysis of the covariate of age due to surgical ovariectomy at a relatively young age.

We next evaluated fixed effects (treatment) and random effect covariates using the linear mixed-effects model. Covariates included age: under 52 years versus over 52 years; education: 16 years versus more than 16 years; MHT using systemic estradiol treatment within the last 5 years (yes versus no); as well as estriol dose (2mg versus more than 2mg). Significant improvement from pretreatment baseline month 0 to treatment month 12 was again detected using this analysis in all six cognitive symptoms: Brain Fog (p = 6.25e-09), Concentration (p = 2.48e-06), Working Memory (p = 1.80e-07), Processing Speed (p = 8.37e-08), Verbal Memory (p = 7.49e-08), and Problem Solving (p = 2.33e-04). Patients were then separated into two subgroups in each random effect covariate (Fig. 2). All subgroups showed improvement from month 0 to month 12. Regarding baseline differences, there were none other than the effect of education on working memory. Subjects with more than 16 years of education reported less severity in working memory issues than those with 16 years of education (p = 0.007, Welch’s Two-Sample t-test). To determine change from month 0 to month 12 in each subgroup, Welch’s t-test was performed on slopes from linear regression models for each subgroup. Extracted slopes and standard errors were used. None of the slopes were significantly different between subgroups. Thus, from pretreatment baseline month 0 to treatment month 12, there was improvement in cognitive domain-specific symptoms in menopausal women regardless of age, education level, or prior MHT use. Regarding estriol dose, symptoms improved from pretreatment baseline month 0 to treatment month 12 in those taking estriol at the 2mg dose and in those taking more than 2mg.

### Estriol treatment improves cognitive function and reverses hippocampal neuropathology in estrogen deficient female mice at midlife

Mouse models are used almost universally to study biologic mechanisms in humans. The mouse model chosen is based on the question asked, focusing on a particular aspect of human disease. Even though a mouse model cannot recapitulate all aspects of human disease, it informs mechanistic questions in humans. Cognitive testing in mice is extensively used by neuroscientists to discover mechanisms involving cognition in humans. Finally, therapeutic drug development and screening routinely include treatments in mice, since this reveals the effect of treatment on regional neuropathology and the effect of selective gene deletion on treatment efficacy.

Here, we used the previously established preclinical model of cognitive deficits and neuropathology of menopause, one where an interaction between midlife aging (months 12-14) and complete estrogen deficiency (induced by ovariectomy) was shown to induce cognitive deficits and dorsal hippocampal atrophy on *in vivo* MRI, with glial activation and synaptic loss therein^17^. This is an informative model since menopausal women studied longitudinally have shown hippocampal atrophy and risk of cognitive decline^13,67^.

**Fig. 3:**
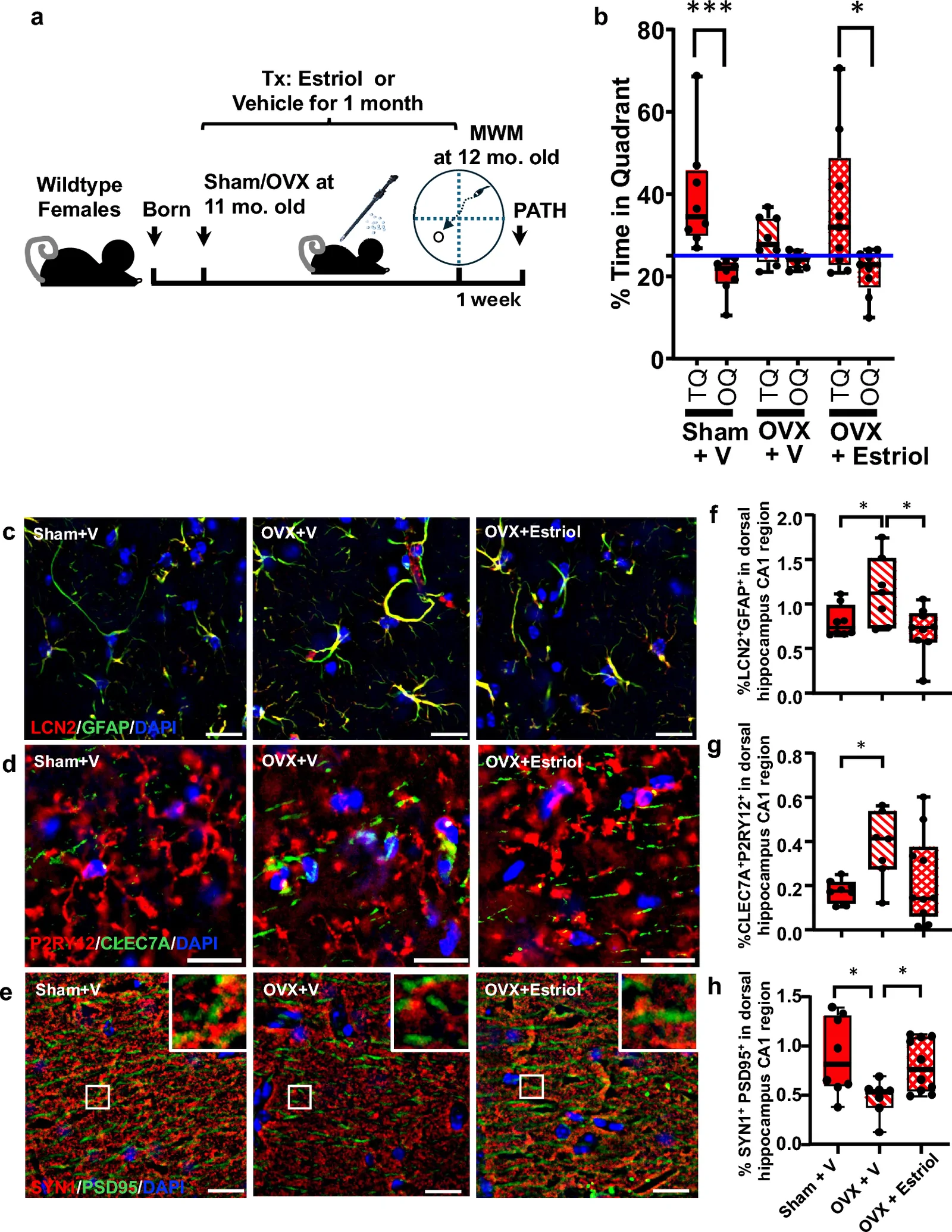
Estriol treatment improves cognitive function and neuropathology in the menopausal model. (**a**) Schematic showing timing of ovariectomy (OVX) or sham surgery, estriol or vehicle (V) treatment, Morris Water Maze testing (MWM), and pathology (PATH) at midlife age. (**b**) Gonadally intact vehicle-treated midlife females (Sham + V, solid), ovariectomized vehicle-treated midlife females (OVX + V, diagonal lines) or ovariectomized E3-treated midlife females (OVX + Estriol, crossed lines) were assessed for spatial reference memory by MWM. A probe trial was conducted 2 h after the 5-day platform hidden training (without escape platform). % of time in the target quadrant of the maze (TQ) indicated memory of the platform location in preference to the average of other 3 quadrants (OQ). Gonadally intact vehicle-treated mice showed preference for the TQ over the OQ (*p* = 0.0003), while ovariectomized vehicle-treated did not. In contrast, ovariectomized estriol-treated restored preference for the TQ over the OQ (*p* = 0.039). N=8-9. **(c-f)** Representative ×40 images of (c) LCN2 (red), GFAP (green), merged images for colocalization (yellow) showing astrocyte reactivity, (d) CLEC7A (green), P2RY12 (red), merged image for colocalization (yellow) showing disease-associated microglia (DAM) , and (e) SYN1(red), PSD95 (green), merged images for colocalization (yellow) showing pre- and post-synaptic staining. Nuclei were counterstained with DAPI (blue). Bar = 20 um Corresponding ×10 magnification images are shown in Fig. S2c-e. (**f-h**). Quantitative analysis of (f) LCN2^+^GFAP^+^ area fraction for astrocyte reactivity. CLEC7A^+^P2RY12^+^ area fraction for disease-associated microglia (DAM), and (h) SYN1^+^ PSD95^+^ area fraction for pre-, and post-synapses each in dorsal hippocampus CA1 region in sham + V, OVX + V, and OVX + Estriol. Estriol treatment prevented the increase in reactive astrocytes (p= 0.044) and loss of synapses (p= 0.018) in midlife OVX females, with a trend of reduced disease-associated microglia (p= 0.071). Area fraction represents the percentage of the field of view occupied by co-localized signal. N=7-10. All box plots with center lines showing the medians, boxes indicating the interquartile range, and whiskers indicating from the minimum and to the maximum values. Two-sided Welch’s t-tests applied to rank-transformed data with Benjamini–Hochberg correction for multiple comparisons. *p < 0.05, **p< 0.01, ***p< 0.001.

Midlife ovariectomized (OVX) wild-type female were treated for one month with either estriol or vehicle. The estriol dose had previously been shown to be neuroprotective in MS preclinical models and to induce a blood concentration consistent with late pregnancy estriol levels in healthy mice. Outcome measures included cognitive testing using Morris water maze (MWM) and dorsal hippocampal neuropathology (see Schematic, Fig. 3a). As expected, MWM behavioral testing showed intact cognitive performance in midlife sham-operated, vehicle-treated, female mice, as indicated by a significant preference for the target quadrant (TQ) over the average of other quadrants (OQ) (Hodges-Lehmann median difference (MD)= -15.03, 95% Confidence Intervals (CI) [-25.61, -8.278], p=0.0003; Fig. 3b). In contrast, OVX, vehicle-treated midlife female mice had a cognitive deficit, as indicated by no significant preference for the TQ compared to OQ (MD =-4.494, 95% CI [-10.53, 0.050], p=0.076; Fig. 3b). Importantly, OVX female mice at midlife that were treated with estriol demonstrated intact cognition, as shown by a significant preference for the TQ over OQ (MD =-10.16, 95% CI [-29.43, -0.594], p=0.039; Fig. 3b). There were no between groups differences in training (Fig. S2a) or swim speed (Fig. S2b). Thus, differences in MWM performance were not due to differences in the ability to learn, they were due to differences in the ability to remember.

Since we had previously shown that cognitive function in the menopause model correlates with dorsal hippocampal atrophy by MRI and that neuropathology occurs therein ^8^, we next determined the effect of estriol treatment on neuropathology in the dorsal hippocampus. We focused on the CA1 region since it is involved in spatial reference memory^68–70^, thereby aligning with the cognitive testing used here and previously^17^. In addition, the CA1 is known to be sensitive to hormonal modulation, including estrogen signaling^57,71^. Reactive astrocytes were assessed by colocalization of LCN2 and GFAP (Fig. 3c), disease-associated microglia by colocalization of CLEC7A and P2RY12 (Fig. 3d), and synaptic loss by assessing pre- and post - synaptic markers SYN1 and PSD95, respectively (Fig. 3e). Midlife OVX-vehicle-treated female mice demonstrated significantly increased reactive astrocytes (MD =0.303, 95% CI [0.014, 0.714], p= 0.046; Fig. 3f), increased disease-associated microglia (MD =0.214, 95% CI [0.063, 0.356 ], p= 0.032; Fig. 3g), and reduced synaptic staining (MD = -0.429, 95% CI [ -0.843, -0.030 ], p= 0.018; Fig. 3h), each compared to midlife sham-vehicle-treated females, consistent with observations previously reported^17^. Conversely, estriol treatment of midlife OVX females reduced reactive astrocytes MD = -0.39, 95% CI [-0.83, -0.02], p = 0.044; Fig. 3c,f) and increased synapses (MD = 0.36, 95% CI [0.01, 0.57], p = 0.018; Fig. 3e,h), restoring levels to those observed in sham-operated control midlife mice, with a trend for reduction in disease-associated microglia (MD = -0.18, 95% CI [-0.39, 0.04], p = 0.071; Fig. 3d,g). Together, these results demonstrate that estriol treatment improves cognitive performance and reduces hippocampal neuropathology in a preclinical model of cognitive deficits of menopause.

### Increased ENO1 protein expression in dorsal hippocampal reactive astrocytes in estrogen deficient female mice at midlife correlates with cognitive impairment

**Fig. 4:**
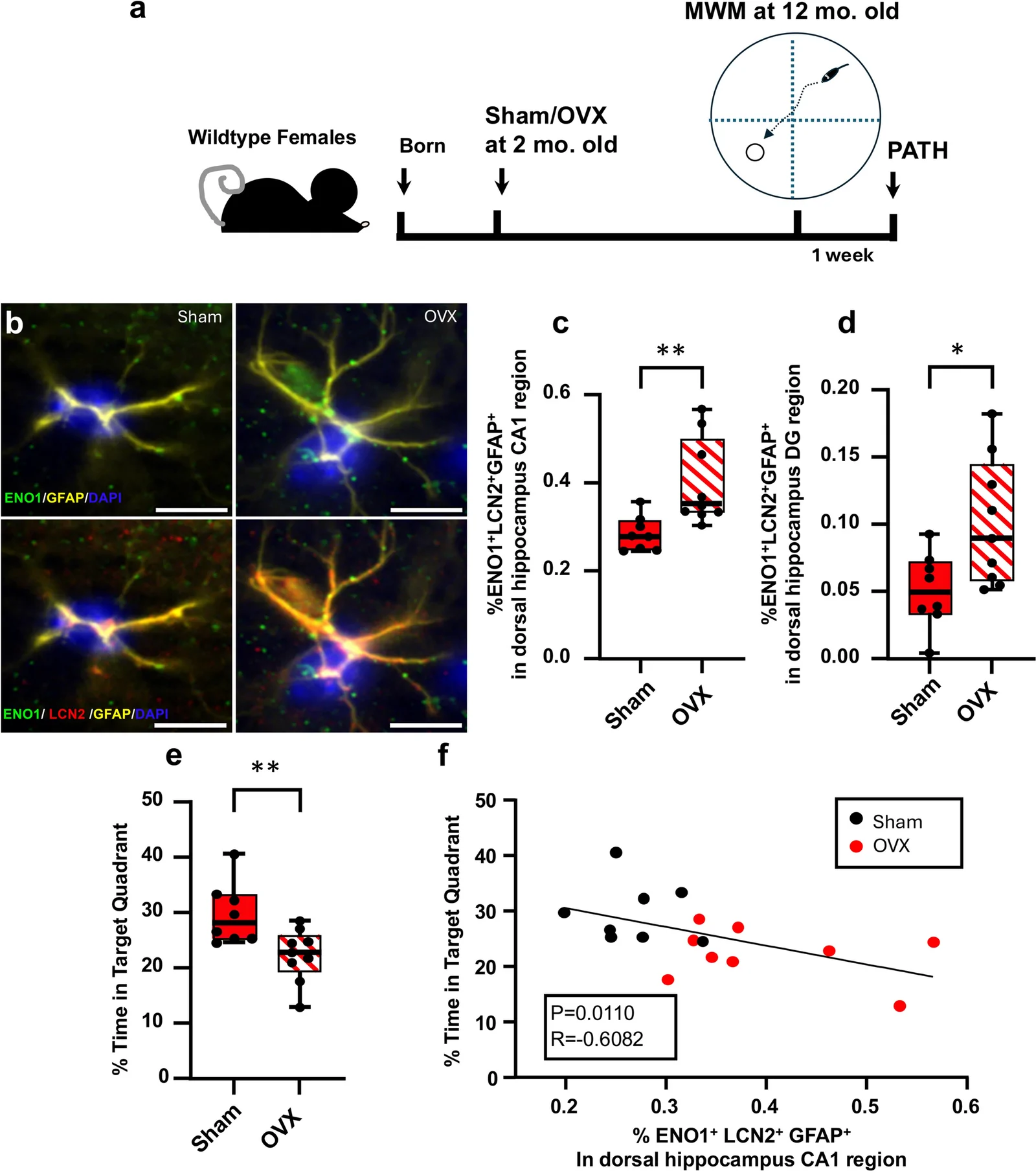
Estrogen loss increases ENO1 protein at midlife in hippocampal astrocytes which correlates with cognitive impairment. (**a**) Schematic showing timing of ovariectomy (OVX) or sham surgery, cognitive testing (MWM), and pathology (PATH) at midlife age. (**b**) Representative images of ENO1^+^ (green), LCN2^+^ (red), GFAP^+^ (yellow), and merged images in the dorsal hippocampus. Nuclei stained with DAPI (blue). Bar = 10 um. Corresponding 40× magnification images are shown in Fig. S3a. **(c-d)** Quantitative analysis of ENO1^+^ LCN2^+^ GFAP^+^ area fraction in dorsal hippocampus CA1 region (c) and DG (d) in sham and OVX. OVX females (diagonal lines) showed increased ENO1 expression in reactive astrocytes in CA1 (p= 0.0013) and DG (p= 0.029) as compared to the Sham females (solid). Area fraction represents the percentage of the field of view occupied by tri-localized signal. N=8-9. (**e**) MWM cognitive testing. OVX females (diagonal lines) showed a decrease in % time in target quadrant (p= 0.0044), compared to sham females (solid) at midlife age. N=8-9. All box plots with center lines showing the medians, boxes indicating the interquartile range, and whiskers indicating from the minimum and to the maximum values. Two-sided Welch’s t-tests applied to rank-transformed data. *p < 0.05, **p< 0.01. (**f**) Correlation of MWM cognitive performance with ENO1 expression in reactive astrocytes. % Time in target quadrant was negatively correlated with ENO1^+^ LCN2^+^ GFAP^+^ area fraction in dorsal hippocampus CA1 region (r=-0.6082, p=0.0110). N=8-9. Spearman’s correlation analyses.

Previously, selective deletion of ERβ in astrocytes of gonadally intact female mice at midlife recapitulated the deleterious effects of ovariectomy when assessing dorsal hippocampal outcome measures (atrophy by MRI, glial activation and synaptic loss by neuropathology)^17^. Transcriptomic analyses revealed that the most differentially expressed gene pathways in astrocytes were those for glucose utilization (glycolysis and gluconeogenesis). *Enolase 1* (*Eno1*) was the most differentially expressed RNA in these pathways when comparing hippocampal astrocytes from astrocyte ERβ conditional knockout (cKO) mice versus age-matched wildtype littermate female controls^17^. Here, in the menopause model, ENO1 expression was determined at the protein level in dorsal hippocampal reactive astrocytes, and its relationship to cognitive performance was shown. OVX or sham surgery was performed and immunostaining for ENO1 protein was done (see Schematic, Fig. 4a). Results showed an increased ENO1 protein expression in reactive astrocytes (tri-localized ENO1^+^LCN2^+^GFAP^+^ area) in the dorsal hippocampal CA1 (MD = 0.085, 95% CI [0.033, 0.217] , p = 0.0013; Fig. 4b - c) and dentate gyrus (DG) regions (MD = 0.045, 95% CI [0.0043, 0.093] , p = 0.029; Fig. 4d) of OVX female midlife mice, each as compared to sham controls. OVX midlife females showed significantly worse cognitive performance using MWM testing, namely less percent time spent in TQ (MD = -6.6, 95% CI [-12.40, -1.700], p = 0.0044; Fig. 4e). This memory deficit was not confounded by differences in spatial learning ability (Fig. S3c) or swim speed (Fig. S3d). Further, worse cognitive performance correlated with higher ENO1 expression in hippocampal reactive astrocytes (r = -0.6082, 95% CI -0.847, -0.165], p= 0.011; Fig. 4f). Taken together, the loss of endogenous female hormones increases ENO1 protein expression in dorsal hippocampal reactive astrocytes, which aligns with impaired cognitive test performance at midlife. Regarding relevance to humans, previously gene expression analyses using existing datasets from autopsy brain tissues showed that ENO1 expression in brain was higher in women over age 60, compared to women under age 50 years^17^.

### Increased ENO1 protein expression in midlife female mice with selective deletion of ERβ in astrocytes correlates with cognitive impairment

**Fig. 5:**
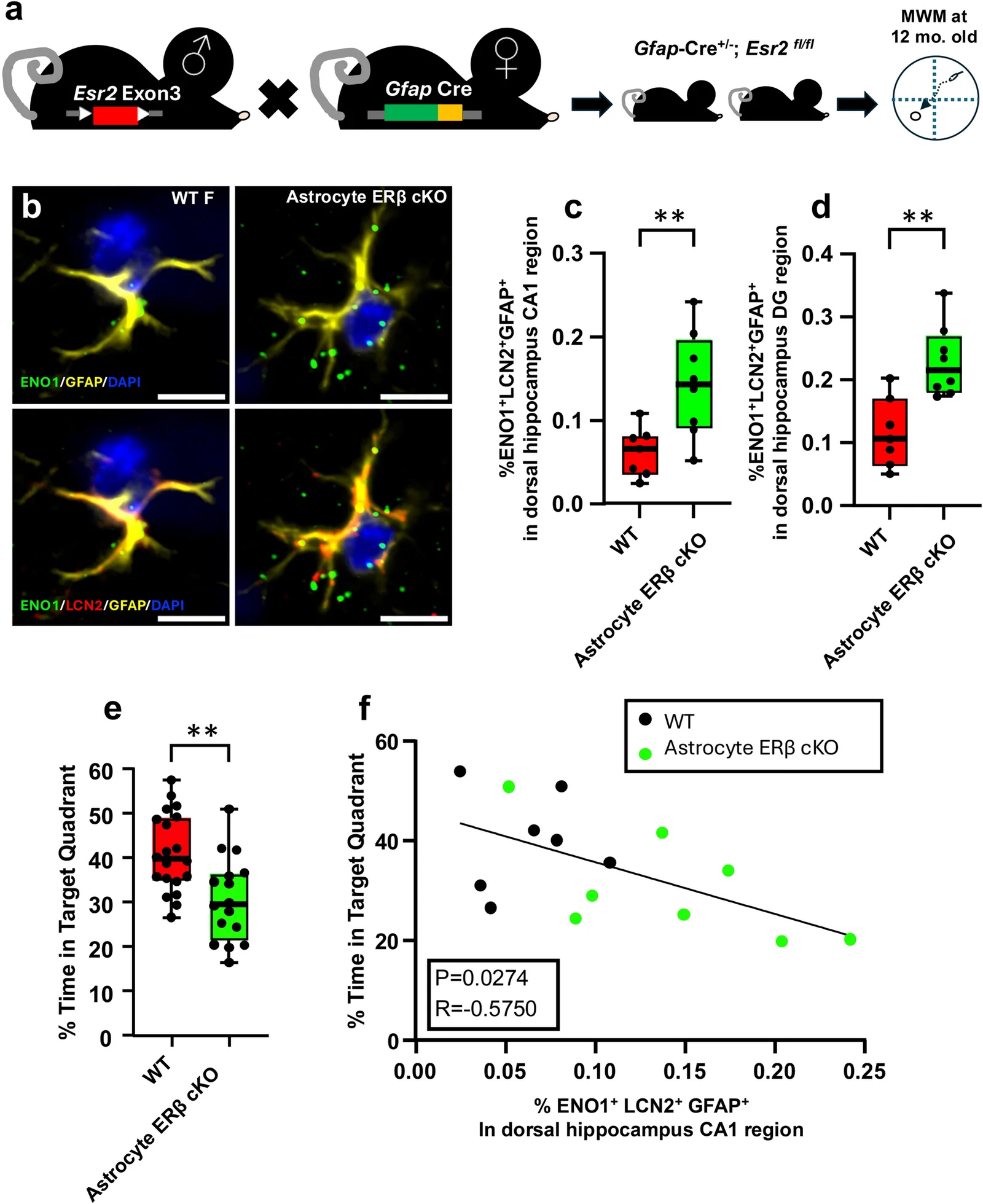
Selective deletion of ERβ in astrocyte increases ENO1 protein in hippocampal astrocytes which correlates with cognitive impairment. (**a**) Breeding scheme to generate *mGFAP-Cre(77.6); ERβ*^*fl/fl*^ mice. Offspring underwent cognitive testing (MWM), and pathology at midlife age. (**b**) Representative images of ENO1^+^ (green), LCN2^+^ (red), GFAP^+^ (yellow), and merged images for tri-localization (orange) showing ENO1 expression in reactive astrocytes in the dorsal hippocampus CA1 region. Nuclei stained with DAPI (blue). Bar = 10 um. Corresponding 40× magnification images are shown in Fig. S4a. (**c-d**) Quantitative analysis of ENO1^+^ LCN2^+^ GFAP^+^ area fraction in dorsal hippocampus CA1 region (c) and dentate gyrus (DG, d) in WT (red) and astrocyte ERβ cKO (green). ENO1 protein expression in astrocyte ERβ cKO was increased in reactive astrocytes at CA1 (p= 0.0049) and DG (p= 0.0019), compared to the WT females. Area fraction represents the percentage of the field of view occupied by tri-localized signal. N=7-8. (**e**) MWM cognitive testing. Astrocyte ERβ cKO females showed a decrease in % time in target quadrant (p= 0.0018), compared to littermate-wildtype (WT) females at midlife. All box plots with center lines showing the medians, boxes indicating the interquartile range, and whiskers indicating from the minimum and to the maximum values. Two-sided Welch’s t-tests applied to rank-transformed data. **p< 0.01. (**f**) % Time in target quadrant was negatively correlated with ENO1^+^ LCN2^+^ GFAP^+^ area fraction in dorsal hippocampus CA1 region (r= -0.575, p= 0.027). N=7-8. Spearman correlation analyses.

Next, we determined whether selective deletion of ERβ in astrocytes in gonadally intact females at midlife alters ENO1 protein expression in dorsal hippocampal reactive astrocytes. To this end, the *mGFAP-Cre 77.6* line was crossed with mice containing homozygous floxed *Esr2* alleles (see Breeding scheme, Fig. 5a). Validation of ERβ deletion specifically in astrocytes of hippocampus was previously published^17^. Since the objective of selective deletion of ERβ in astrocytes was to determine if physiologic levels of ovarian hormones act on ER beta in astrocytes to confer neuroprotection in midlife females, OVX was not done.

Significant increases in ENO1 protein expression were observed within dorsal hippocampal CA1 (MD = 0.0724, 95% CI [0.017, 0.138], p = 0.0049; Fig. 5b-c) and DG regions (MD = 0.111, 95% CI [0.045, 0.182] , p = 0.0019; Fig. 5d) in astrocyte ERβ cKO females at midlife compared to age matched wildtype (WT) female controls. Midlife astrocyte ERβ cKO female mice also had impaired cognitive function as shown by reduced % time spent in TQ, compared to WT littermate controls (MD = -10.56, 95% CI [-16.88, -4.200], p = 0.0018; Fig. 5e). This impairment was not confounded by differences in spatial learning ability (Fig. S4b) or swim speed (Fig. S4c). Further, increased ENO1 expression in hippocampal reactive astrocytes correlated with worse cognitive performance (r= -0.575, 95% CI [-0.845, -0.072], p= 0.027; Fig. 5f). These results demonstrate that the selective deletion of ERβ in astrocytes at midlife increases ENO1 expression in dorsal hippocampal reactive astrocytes, and this upregulation is associated with impaired cognitive test performance. Together, this reveals the critical role of ERβ in astrocytes for cognitive function in midlife females.

### ERβ-ligand treatment of midlife females reverses the increase in ENO1 expression in dorsal hippocampal reactive astrocytes induced by OVX

**Fig. 6:**
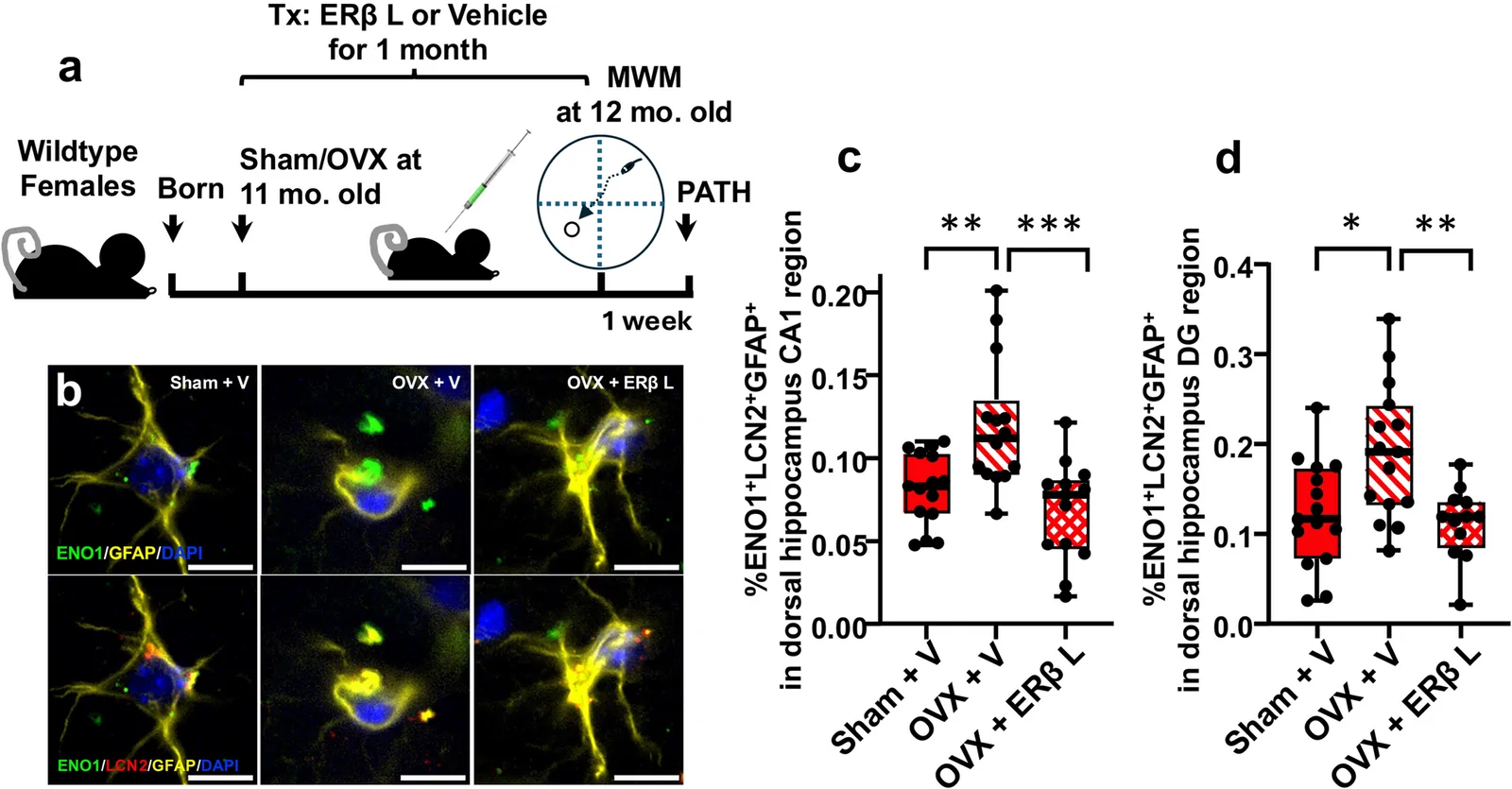
ERβ-ligand treatment decreases ENO1 expression in hippocampal astrocytes of midlife OVX female mice. (**a**) Schematic showing timing of ovariectomy (OVX) or sham surgery, ERβ ligand (ERβ L) or vehicle (V) treatment, cognitive testing (MWM), and pathology (PATH) at midlife age. (**b**) Representative images of ENO1^+^ (green), LCN2^+^ (red), GFAP^+^ (yellow), and merged images in the dorsal hippocampus. Nuclei stained with DAPI (blue). Bar = 10 um. Corresponding 40× magnification images are shown in Fig. S5. (**c-d**) Quantitative analysis of ENO1^+^ LCN2^+^ GFAP^+^ area fraction in dorsal hippocampus CA1 region (c) and DG (d). OVX + ERβ L (crossed lines) showed decreased ENO1 expression in reactive astrocytes in CA1 (p= 0.0004) and DG (p= 0.008) as compared to the OVX + V group (diagonal lines). Area fraction represents the percentage of the field of view occupied by tri-localized signal. N=12-15. All box plots with center lines showing the medians, boxes indicating the interquartile range, and whiskers indicating from the minimum and to the maximum values. Two-sided Welch’s t-tests applied to rank-transformed data with Benjamini–Hochberg correction for multiple comparisons. *p < 0.05, **p< 0.01, ***p<0.001.

Since “loss-of-function” studies showed that selective deletion of ERβ in astrocytes in gonadally intact midlife females caused increased ENO1 protein expression in hippocampus, we next determined whether “add back” pharmacological activation of ERβ could reverse this. WT females were OVX and treated with either an ERβ ligand (diarylpropionitrile, DPN) or vehicle, followed by MWM behavioral testing and dorsal hippocampal neuropathology^17^ (see Schematic, Fig. 6a). The protective effect of the ERβ ligand treatment on MWM testing in this model was previously published^17^. Here, these OVX-vehicle-treated females showed increased ENO1 expression (tri-localized ENO1^+^LCN2^+^GFAP^+^ area) in the dorsal CA1 (MD = 0.030, 95% CI [0.009, 0.058], p = 0.0019; Fig. 6b–c) and DG regions (MD = 0.067, 95% CI [0.012, 0.117], p = 0.017; Fig. 6d) compared to sham-vehicle-treated females. Conversely, this ENO1 upregulation was reversed in OVX midlife females treated with the ERβ ligand (CA1: MD = -0.044, 95% CI [-0.077, -0.017], p = 0.0004; Fig. 6c, DG: MD = -0.073, 95% CI [-0.125, -0.018], p = 0.008; Fig. 6d), indeed restoring low levels like those observed in controls (sham-operated).

### Estriol treatment of midlife females reverses the increase in ENO1 expression in dorsal hippocampal reactive astrocytes induced by OVX

**Fig. 7:**
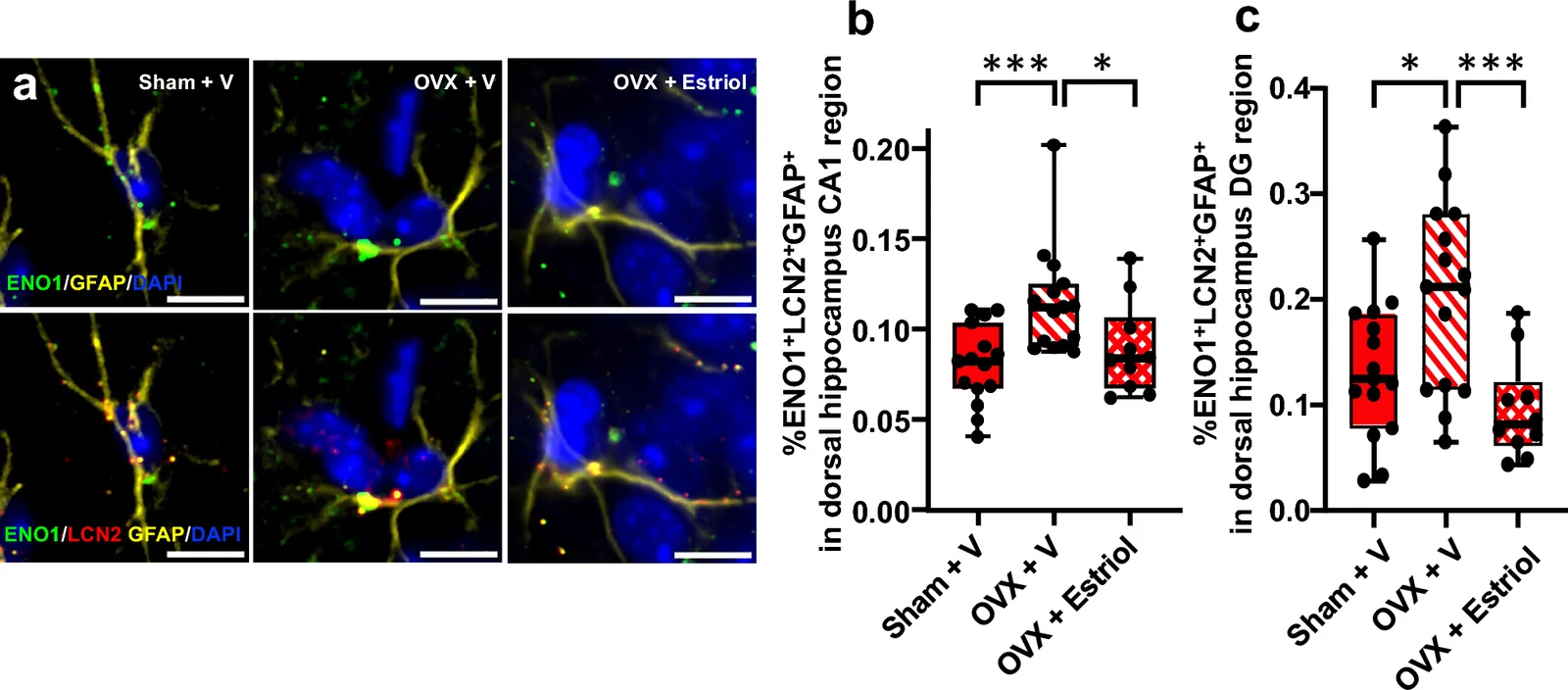
Estriol treatment decreases ENO1 expression in hippocampal astrocytes of midlife OVX female mice. (**a**) Representative images of ENO1^+^ (green), LCN2^+^ (red), GFAP^+^ (yellow), and merged images in the dorsal hippocampus. Nuclei stained with DAPI (blue). Bar = 10 um. Corresponding 40× magnification images are shown in Fig. S6. (**b-c**) Quantitative analysis of ENO1^+^ LCN2^+^ GFAP^+^ area fraction in dorsal hippocampus CA1 region (c) and DG (d). OVX + Estriol (crossed lines) showed decreased ENO1 expression in reactive astrocytes in CA1 (p= 0.015) and DG (p= 0.0008) as compared to the OVX + V group (diagonal lines). Area fraction represents the percentage of the field of view occupied by tri-localized signal. N=10-15. All box plots with center lines showing the medians, boxes indicating the interquartile range, and whiskers indicating from the minimum and to the maximum values. Two-sided Welch’s t-tests applied to rank-transformed data with Benjamini–Hochberg correction for multiple comparisons. *p < 0.05, ***p<0.001.

Finally, estriol, the pregnancy-associated estrogen with preferential binding to ERβ^72^, was tested for its ability to normalize ENO1 expression in hippocampal astrocytes (see Schematic, Fig. 3a). Consistent with findings in previous experiments, OVX-vehicle-treated midlife females had increased ENO1 expression in astrocytes within the dorsal CA1 (MD = 0.029, 95% CI [0.0094 to 0.045], p = 0.0002; Fig. 7a–b) and DG regions (MD = 0.081, 95% CI [0.0056, 0.138], p = 0.029; Fig. 7c). Consistent with observations using ERβ ligand treatment in Fig. 6, targeting ERβ using estriol treatment also reversed ENO1 upregulation induced by OVX (CA1: MD = -0.025, 95% CI [-0.046 to -0.005], p = 0.015; Fig. 7b, DG: MD = -0.115, 95% CI [-0.176 to -0.038], p = 0.0008; Fig. 7c), indeed normalizing levels to those observed in hippocampal astrocytes of controls (sham-operated). Together, these results demonstrate a mechanism of action for estriol treatment-mediated beneficial effects on cognitive performance and hippocampus pathology using the preclinical model of cognitive deficits of menopause.

## Discussion

Menopause affects multiple organs beyond gynecology, including brain, bone, heart, metabolism, colon, and skin. As Comprehensive Menopause Centers emerge, and gynecologists collaborate closely with teams of experts in other specialties, the challenge is to treat a vast array of deleterious effects caused by being estrogen deficient for at least a third of one’s life (ages early 50s to 80s). While not all aspects of aging can be prevented, those due to loss of estrogen and progesterone during menopause could be. There is an urgent need to develop MHT strategies that go beyond reducing vasomotor symptoms of perimenopause and early menopause. Development of an MHT tailored for long term protection of each organ is needed. A foundation for optimally tailored MHT discovery can be based on functional activity of ERα versus ERβ in each organ, since these ERs are known to have synergistic or antagonistic effects depending on cell and tissue type^42,43,73^.

This case series was designed to explore a candidate treatment targeting cognitive domain-specific symptoms of menopause. This addresses a major unmet need, since cognitive complaints are reported in most menopausal women, an eventual concern in nearly half the population. Menopause related regional changes in brain imaging further underscore the need for an MHT that is directly neuroprotective. Pearlpak treatment is a blisterpack designed to target specific brain cells and processes involved in cognition. It repurposes estriol’s neuroprotective effects in another neurodegenerative condition, namely women with MS. Pearlpak was designed to ensure compliance of dosing and timing in a population that has memory issues. Compliance matters, since higher blood estriol levels previously correlated with better processing speed scores in MS women randomized to a single estriol dose^64,65^. Here, the case series in menopausal women showed significant reductions in cognitive domain-specific symptoms after 12 months of treatment, the same timepoint that significant benefits were previously reported in MS women for improvement in processing speed^49,65^, reduction in cerebral cortex atrophy, and decreased serum neurofilament light chain^61,62,64,65^.

Limitations include those inherent to case studies in human subjects, namely the small sample size and lack of a placebo control. Also, all subjects had a college degree or higher, making it unclear whether cognitive symptom improvement would be stronger or weaker in the general population. Future comparator-controlled, randomized trials are warranted that include brain imaging for regional changes and formal cognitive-domain specific longitudinal testing, as well as apolipoprotein E (APOE)-e4 genotype testing^12,13^ and quantification of other menopause outcomes (vasomotor symptoms, sleep, anxiety, and depression)^1^. A formal dose finding trial would also be informative.

In research that is translational between women with menopause and an animal model of menopause, one should consider the effect of age. This bears relevance to the “timing hypothesis” of estrogen deficiency and estrogen treatment^74–79^. In our previous paper describing a mouse model of menopause, there was complete loss of estrogen induced by ovariectomy at 2 months of age with cognitive outcomes assessed in young mice (4 months), midlife mice (12-14 months), and old age mice (20-22 months)^17^. Females at old age, with or without ovariectomy at 2 months, showed end stage effects on dorsal hippocampal atrophy. In contrast, midlife females who did not have prior ovariectomy did not have dorsal hippocampal atrophy. Thus, aging to midlife alone did not cause dorsal hippocampal atrophy. However, midlife females with prior ovariectomy did exhibit atrophy, indeed approximating that observed in old age. This discovery was the previously described “sex hormone by age interaction”, namely that the combination of midlife aging with long-term loss of estrogen is deleterious. Here, we extended these studies using ovariectomy at either 2 months or 11 months of age, again examining cognitive outcomes and treatment effects at midlife. Our focus was to determine mechanisms that occur before midlife to prevent cognitive issues at midlife.

Distinct from the effect of age, one can view estrogen deficiency as having acute and chronic effects. Cognitive issues of menopause in women can occur with acute changes in ovarian hormones during perimenopause, and this has been associated with hot flashes, night sweats, poor sleep, and changes in mood^5,80^. Perimenopausal fluctuations in ovarian hormones usually start at 45-47 years of age, but can start as early as age 40. The mean age of menopause is 51.5 years (range 48 to 54 years). In addition to cognitive issues due to acute loss in ovarian hormones, cognitive issues can be due to chronic loss of ovarian hormones as shown by cognitive symptoms in women who never had hot flashes or in women age 60 with no hot flashes for years. Conversely, regional brain protection has been reported in women with more years of estrogen exposure (more time between menarche and menopause) or more exposure to high levels of estrogen during pregnancy (multiparity). Long term effects of being estrogen deficient are now being mapped by longitudinal clinical research that includes both cognitive domain-specific testing and brain region-specific neuroimaging^12,13^. For context, there are chronic effects of being without ovarian hormones not only for the brain, but also for the genitourinary system, bone, heart, metabolism, colon, and skin. Comprehensive menopause clinics manage both acute and chronic symptoms, which are not mutually exclusive. They start mainly as acute effects, then transition to primarily chronic effects, with overlap as they evolve. Our focus is to disentangle mechanisms underlying long-term effects of estrogen deficiency on cognition and to prevent these long-term effects by using treatments targeting specific estrogen receptors in brain cells.

In the menopause model used here, complete estrogen deficiency and midlife aging combined to cause worse cognitive test performance, hippocampal atrophy, glia activation, abnormal glucose utilization in astrocytes, and synaptic loss^17^. Increased ENO1 of the Glycogenesis and Gluconeogenesis Pathways was discovered during RNA sequencing of the astrocyte specific transcriptome^17^. Findings here showed that ENO1 expression was increased at the protein level in astrocytes from OVX wild type mice and in gonadally intact mice with selective deletion of ERβ in astrocytes. Higher ENO1 expression in astrocytes correlated with worse performance in cognitive testing. We previously reported that menopausal age women have higher ENO1 levels in hippocampus^17^, and others reported that^18^FDG-PET shows hypometabolism of glucose during the menopause transition^8,14^. While ENO1 is traditionally a facilitator of glycolysis by providing essential lactate to neurons, the estrogen-deficient brain may upregulate ENO1 as an attempt to compensate for a pathological metabolic shift during menopause. Since astrocytes maintain synaptic health^81^, we hypothesize that poor glucose utilization by astrocytes during menopause may contribute to synaptic dysfunction and synaptic loss in aging women.

Limitations in the mouse experiments are that there could be differences between ovariectomy at 2 months of age versus 11 months of age. Answering this question is beyond scope here as it will require a head-to-head comparison between mice ovariectomized at 2 months (with sham control) versus 11 months (with sham control), followed by Morris Water Maze testing and dorsal hippocampal pathology assessed in parallel at 12 months, all within the same experiment to permit statistical comparisons between groups. Here, we assessed outcomes independently in separate experiments that were focused on different objectives. Thus, we cannot draw conclusions regarding possible significant differences in the effect of OVX at age 2 months vs OVX at age 11 months, when our outcomes are assessed at 12 months. Also, while the Morris Water Maze is widely used to assess dorsal hippocampal function, namely spatial memory, there may be other cognitive domains of interest. To that end, a composite score of multiple cognitive tests of various brain regions is now warranted.

Together preclinical and clinical findings suggest the following mechanism. Estriol treatment induces detectable blood levels of estriol, and systemic estriol is known to cross the blood brain barrier. Estriol binds to ERβ in hippocampal astrocytes, to reduce astrocyte and microglial activation, normalize brain glucose utilization in astrocytes, and decrease synaptic loss. This improves hippocampal-dependent cognition symptoms induced by loss of neuroprotective estrogen during menopause.

Direct and indirect effects of estriol treatment on cognition can both occur.. Some subjects in this case series had vasomotor symptoms, while others did not. While we did not quantify vasomotor symptoms in this study, younger menopausal women are known to have vasomotor symptoms more often than older menopausal women, since hot flashes usually dissipate over the first three to seven years after perimenopause. Direct benefits of estriol treatment on cognition through binding to ERβ in cells of the brain^17,58,82^ are not mutually exclusive of indirect benefits of estriol treatment through its ability to decrease hot flashes and night sweats, thereby improving sleep for better cognition the next day. Notably, not all subjects here had vasomotor symptoms, yet all reported cognitive improvement. Also, MS women treated in clinical trials with estriol were younger and very few had vasomotor symptoms, yet they demonstrated improvement in cognition, regional brain atrophy, and a blood biomarker of neurodegeneration in clinical trials^49,61,62,64,65^.

Menopausal women must not take “unopposed estrogen” if they have a uterus, since progesterone protects from uterine endometrial hyperplasia. Most women here took progesterone since most had a uterus. It was micronized progesterone, not medroxyprogesterone of the past Women’s Health Initiative^83–85^. Progesterone is known to have sedative properties, so is taken at night. This avoids sedative effects during the day and helps with difficulty sleeping at night. Neuroprotective effects of progesterone in demyelinating models of MS and in cerebral ischemia models of stroke have been described^86–89^. It remains unknown whether there are significant differences in the effect on cognition in menopausal women taking estriol alone versus the combination of estriol and progesterone.

Finally, what about repurposing a neuroprotective treatment from one neurodegenerative disease that predominantly affects women, namely MS, to another, namely AD? Previously, menopause-induced cognitive issues and brain imaging abnormalities were not associated with later development of Mild Cognitive Impairment (MCI) or AD in humans^1^. However, past studies did not determine scores on cognitive domain-specific testing alongside changes in hippocampal volumes and other neuroimaging outcomes in otherwise healthy menopausal women followed longitudinally from ages 50 to 75 years. This is needed, since two thirds of AD patients are women, and treatments for AD are thought to be more efficacious at the earlier stage of MCI^12,13^. This study is now ongoing. While longitudinal data are being accrued, cross-sectional analyses of baseline data from large numbers of women in the UK Biobank and the European Prevention of Alzheimer’s Dementia cohort suggested that APOE genotype and age of MHT initiation may affect hippocampal volumes and possibly cognitive domain-specific test performance^12,13^. Unfortunately, MHT treatments were variable and self-reported regarding age of initiation and duration, with no information on the estrogen or progesterone type, dose, or compliance. Beyond these future longitudinal correlations, causality can only be established through an intervention with subsequent determination of effect, and unfortunately there is no FDA or EMA approved intervention to treat cognitive issues of menopause. To this end, Pearlpak now warrants investigation as such an intervention. Initiating treatment early as a preventative treatment approach, before AD onset, will be likely be very important based on studies of estrogen receptor expression in autopsy tissues of women with AD. ERα expression in hippocampal tissues from women with AD was decreased^90^. Also, frontal cortex showed reduced ERβ in mitochondria of neurons, and this was thought to contribute to the mitochondrial dysfunction involved in AD pathogenesis in women^91^.

In conclusion, the focus of this study is brain health. However, the roadmap of using ER specificity to drive distinct treatments targeted for each organ system is applicable to other organ systems affected by menopause. One can optimize efficacy for the primary indication and minimize toxicity in other organs, such as breast. Comprehensive Menopause Centers are ideally suited with the ability to not only determine the effect of a targeted treatment on one organ system, but also on others, either positive or negative, indeed comprehensively.

## Materials and methods

### Human subjects

Subjects in this case series were treated as part of standard patient care. Natural menopause was defined according to North American Menopause Society (NAMS) as 12 consecutive months of amenorrhea with no alternative etiology. One subject had surgical ovariectomy, and this is stated. Subjects were screened for inclusion based on cognitive symptoms reported to their gynecologist at the University of California, Los Angeles (UCLA) Comprehensive Menopause Program (from September 2023 to January 2025) with in-person or telemedicine visits or by Cleopatrarx with telemedicine visits. Subjects were assessed for standard menopausal replacement therapy (MRT) contraindications by a physician. If there were no contraindications to MRT, they were treated with Pearlpak by Cleopatrarx. There was no randomization and there was no comparator control treatment. Higher estriol doses were used in those with more severe hot flashes. Higher progesterone doses were used in those with more difficulty sleeping. In this case series, standard-of-care was used for dosing, based not only on these two symptoms. Instead, it was individualized for overall health through doctor patient shared decision making. Each subject had follow-up every 3 months (month 0, 3, 6, 9, 12, with more as needed) to assess how they were doing and assure there were no safety issues in accord with clinical standard-of-care. Informed consent was obtained from all subjects acknowledging and agreeing that data may be collected during patient care and that data would be de-identified and aggregated with those of other participants for purposes of research and quality of care improvement and monitoring. All information was handled in accordance with applicable privacy laws, including the Health Insurance Portability and Accountability Act (HIPAA). No personally identifiable information was shared, and individual privacy was fully protected. The study was performed in accordance with the Declaration of Helsinki in its currently applicable version. This study focused on cognitive assessment questionnaires during standard patient care was submitted for IRB review to WCG Clinical. The WCG IRB Board found minimal safety risk and approved an Exemption from full review (#1401370).

### Estriol clinical treatment in menopausal women

All patients received oral estriol each morning for 12 months. To prevent the effect of unopposed estrogen use on the uterine endometrium, patients also received oral progesterone at night, some for 14 days of each 28 day cycle and others every night, based on tailored, individualized clinical care (sleep issues, etc). To increase compliance, hormones were administered in a blisterpack (Pearlpak). Cognitive complaints were quantified using the *Voskuhl Menopause Brain Fog Assessment* at baseline (prior to treatment) and after 12 months of treatment. (Supplemental information).

The *Voskuhl Menopause Brain Fog Assessment* was a questionnaire designed to collect self-reported quantitation of cognitive domain specific symptoms in menopausal women and was based on publications demonstrating which cognitive domains are most affected when menopausal women have undergone cognitive testing by neuropsychologists. The Cognitive Change Index (CCI) and the Montreal Cognitive Assessment (MoCA), which are used for Alzheimer’s Disease (AD) and Mild Cognitive Impairment (MCI), are relatively insensitive in otherwise healthy menopausal women.

Paired samples Wilcoxon signed rank test was used to determine significance of change in cognitive scores during treatment (12 months) compared with pretreatment baseline (month 0). Effect size was calculated using R package “wilcoxonPairedR”. Influence of four covariates on the assessment scores were examined using linear mixed-effects model (R package “lmerTest”) and Welch’s t-test.

### Animals and breeding strategy

All mice used in this study were from the C57BL/6J background. Mice were assigned to groups using a simple randomization procedure independent of body weight. Specifically, animal IDs were randomly assigned to groups prior to interventions. Baseline body weights did not differ between groups compared. We generated mice with selective deletion of ERβ in astrocytes. GFAP-Cre:ERβ^fl/fl^ (astrocyte ERβ cKO) were generated by crossing the GFAP-Cre line (B6.Cg-Tg(Gfap-Cre)77.6Mvs/2J, JAX) with the exon 3 ERβ^fl/fl^ line^17^.

In breeding of our mouse lines, Cre recombinase alleles were always inherited from females (mother), not from males (father). This is because one Cre line showed ectopic expression of Cre recombinase in the male germline (*mGFAP-Cre* 77.6 line; https://www.jax.org/strain/024098). Thus, paternal inheritance of Cre recombinase should be avoided. To this end, our breeding pairs were: GFAP-Cre:ERβ^fl/fl^ females with ERβ^fl/fl^ males (to generate astrocyte ERβ cKO mice).

Genotypes were assessed by PCR using the following primer sequences (5’-3’): ERβ, CTTCTTAGAGGTACGGATCCC and AATCTCTTTGCCTTCCAGAGC; and Cre, GCACTGATTTCGACCAGGTT and GCTAACCAGCGTTTTCGTTC. All mice were housed in a facility with 12-h light/dark cycles with temperature and humidity controlled and were allowed free access to food and water.

All procedures were done in accordance with the guidelines of the National Institutes of Health and the Chancellor’s Animal Research Committee of the University of California, Los Angeles Office for the Protection of Research Subjects. All animal experiments were approved by the Chancellor’s Animal Research Committee of the University of California, Los Angeles Office (ARC#1998-001). For tissue collection, mice were placed under deep isoflurane anesthesia and euthanized by transcardial perfusion with ice-cold 1× PBS, followed by 4% paraformaldehyde. All methods were reported in accordance with ARRIVE guidelines.

### Animals and exclusion criteria

A total of 153 mice (111 from cohorts in Figs. 4–6 and 42 from Figs. 3 & 7) were used in this study. Sample sizes were determined based on effect sizes from previous studies^17^ . To ensure data integrity and compliance with ARRIVE guidelines, exclusion criteria were pre-defined as follows: (1) post-surgical complications, (2) health issues (e.g., ulcerative dermatitis), (3) poor swimming performance in the Morris Water Maze (MWM) (defined as persistent floating or failure to navigate), and (4) technical issues during tissue processing (e.g., damaged sections). A detailed breakdown of animal allocation, attrition, and final sample sizes for each experiment is provided in Supplementary Table S1. All behavioral and histological analyses were performed by investigators blinded to the treatment/genotype groups.

#### Estriol treatment cohort

(Figures 3 and 7): A total of 42 mice were enrolled across two independent experiments (Sham+V: 16, OVX+V: 16, OVX+Estriol: 10). One mouse was lost post-ovariectomy and one was excluded due to dermatitis. For behavioral (MWM), neuropathological analysis, a single representative cohort was used (n=24-25 total). Within this cohort, 3 mice were excluded from MWM analysis due to poor swimming performance. For ENO1 protein analysis, tissues from both experimental cohorts were pooled to maximize statistical power (n=40 total).

#### Menopause model

(Figure 4): In this cohort (N=21 initially), 4 mice were excluded (2 for health, 2 for swimming), leaving n=17 for final analysis.

#### Astrocyte ERβ cKO experiment

(Figure 5): A total of 44 mice were enrolled across two independent cohorts. For MWM testing, both cohorts were used to ensure reproducibility (N=37) after 7 exclusions: 3 for health, 4 for swimming). For neuropathological analysis (ENO1), brains were processed from one cohort only (n=15).

#### ERβ ligand treatment experiment

(Figure 6): A separate cohort of mice (N=46) was utilized for neuropathology. The behavioral characteristics of this cohort have been previously described^17^. Four mice were excluded from the final histological analysis due to lost after surgery (n=1), health issues (n=2), or technical tissue damage (n=2), resulting in a final analyzed size of n=12-15 per group.

### Ovariectomy in the menopause model

Before the procedure, carprofen (1.2 mg/ml per animal) and saline (0.5 ml) were injected subcutaneously. Mice were anesthetized by inhalational anesthesia with isoflurane. The fur above the lateral dorsal back was removed and the skin was sterilized with betadine and alcohol scrubs. For OVX group, bilateral incisions were made into skin and the ovaries were removed. The muscle layer was closed with absorbable suture, and the skin layer was closed with wound clips. For sham operation, bilateral incisions were made into skin, and the skin layer was closed with wound clips without ovary removal.

For postoperative care, amoxicillin (50 mg/ml) was added to the water for 5 days for antibiotics (0.5 mg/ml water) and another dose of carprofen (0.1 ml) was given within 24 h of surgery. Wound clips were removed 7 days later.

Two different ages at ovariectomy were used for two distinct experimental objectives. The first objective focused on a chronic menopause model, whereby ovariectomies (OVX) were performed at 2 months of age, and animals were assessed for outcome measures at 12 months of age. This timing had previously shown an effect of OVX on midlife mice assessed by cognitive testing, dorsal hippocampal atrophy, and neuropathology^17^. Here, these previous findings were extended to address a potential role of Enolase 1 in astrocytes using the same age of OVX with outcomes in midlife mice^17^. The second objective was to determine the effect of treatment. Treatment from Month 2 to Month 12 in mice is not feasible. Repeated estriol/placebo pellet implantation followed by removal and replacement, as well as numerous ERβ/vehicle ligand injections over several months, induce confounding effects of stress, infections, and less reliability of drug delivery. Therefore, to assess the effect of treatment, ovariectomies were performed at 11 months of age, with ERβ ligand or estriol treatment for 1 month duration (with outcomes assessed at 12 months of age).

We did not assess estrous cycles because this has been well established at the age of focus here, 12-14 months^92,93^. Young mice cycle approximately every 3-5 days. At 9 to 12 months of age, females experience prolonged diestrus with longer cycles, with blood estradiol levels mainly at the lowest level of the cycle, before they transition to estrapause where they retain follicles but become anovulatory. Thus, 12 to 14 months is the start of reproductive senescence. Estrapause in mice is not characterized by complete loss of estradiol, which is why we used OVX in our mouse menopause model, which entailed a sex hormone (complete loss of estradiol) by age (midlife) interaction.

### Treatment in the menopause model

For the estriol treatment experiment, either a 90-day release pellet of estriol at a 5 mg dose (cat# NE141, Innovative Research of America) or a placebo pellet (cat# NC111, Innovative Research of America) was implanted subcutaneously near the scruff of the neck a week after ovariectomy. This dose of estriol was previously shown to induce a serum level of estriol approximating late pregnancy in nonpregnant mice^48^**.** For ERβ-ligand treatment we used diarylpropionitrile (DPN). Treatment was initiated 1 week after ovariectomy. Briefly, DPN was dissolved in 100% ethanol and then mixed into 100% Miglyol®812 (CREMER Oleo GmbH & Co. KG) at 1:9 ratio making the final ethanol concentration of 10%. DPN solution (8 mg/kg/day) or vehicle solution (90% Miglyol – 10% ethanol) was given subcutaneously every other day until the end of each experiment. All assessments were done in a blinded fashion with regards to knowledge of treatment randomization.

### Morris Water Maze (MWM)

MWM testing was performed during the light cycle. All the experimental mice were transferred to the behavior testing room at least 30 min before the tests to acclimate to the environment. Temperature and humidity of the experimental rooms were kept at 23 ± 2 °C and 55 ± 5%, respectively. The brightness of the experimental room was kept dim. All animals were habituated to handling for 1–2 min every day for at least a week prior to experimentation. All experimental procedures were in accordance with the Animal Research Committee at the University of California, Los Angeles (ARC#1998-001). The experiments were conducted in the UCLA Behavioral Testing Core Facility and the investigators who performed experiments and analyses were blinded to mouse group.

Mice were trained in hidden platform MWM task to investigate spatial learning and reference memory.^94,95^ The water maze consists of a round pool, 124 cm in diameter and 50 cm deep. In the hidden-platform test, the animal learns the location of the escape platform in relation to the cues in the periphery of the room, the escape platform remains in the same location (in the center of NW, named as Target Quadrant (TQ)) for all training trials and is located 1 cm below the level of water made opaque with non-toxic white tempera paint. Pool temperature was maintained at 23–25 °C by the addition of warm water. On the first day of training, mice are placed on the platform for 30 s before the first trial. If the mouse jumps from the platform, they are returned to the platform for a cumulative 30 s. After 2 min delay, the first day of training sessions begins. Mice undergo 3 training trials a day for 5 days. A pseudorandom order was applied for the starting quadrants within and across training days to prevent the animals from developing a non-spatial, rote strategy, and to control for potential biases. In each training trial, mice are placed in the pool facing to the edge, 5 cm from the wall in the center of one of the three pool quadrants not containing the escape platform, and the latency to finding the platform is recorded. Each trial ends either when the mouse successfully found the platform or after 1 min. At the end of each trial, mice are allowed to remain on the platform or placed on the platform for 5 s before returning to their home cages. On day 5, 2 h after training, the platform is removed for the probe trial. Mice are recorded for 60 s and assessed for how long they spend in each quadrant searching for the platform. All recordings of probe trial are analyzed using TopScan (Clever Sys, Inc., Reston, VA).

### Histological analysis

Standard histological analysis methods were applied as we described previously.^17^

#### Tissue preparation

Mice were deeply anesthetized in isoflurane and perfused transcardially using Masterflex L/S Economy Drive (Cole-Palmer # 7554-80) with Pump Head L/S EASY-LOAD-II SS (Cole-Palmer # 77202-60) with ice-cold 1× PBS at a rate of 5-7mL/min for 5–7 min, followed by 4% paraformaldehyde at a rate of 5-7mL/min for 5 to 7 min. CNS tissues were dissected and submerged in 4% paraformaldehyde overnight at 4 °C, followed by 30% sucrose for >24 h. Brains were embedded in 7.5% gelatin/15% sucrose solution. The gelatin-embedded tissues were submerged in 4% paraformaldehyde overnight at 4 °C, followed by 30% sucrose for >24 h, and stored at −80 °C after flash frozen by dry ice. The 40-μm thick free-floating brain sagittal-sections were prepared using a cryostat (Leica Biosystems) at −20 °C. Tissues were collected serially and stored in 1x PBS with 0.1% sodium azide in 4 °C.

#### Immunohistochemistry

Prior to histological staining, 40-μm thick brain free-floating sections were thoroughly washed with 1X PBS to remove residual sodium azide. Tissues were incubated with 50% methanol/PBS (1:1) at RT, followed by 1xPBS washing. After heat antigen retrieval with heated 10 mM Citric Acid—0.05% Tween 20, pH6.0, all tissue sections were permeabilized with 0.3% Triton-X and 2% normal serum in 1X PBS for 10 min on ice and blocked with 10% normal serum in 1X PBST for 1 h. Tissues were then incubated with primary antibodies in 1xPBST-2% normal goat serum overnight at 4 °C. The next day, tissues were washed and incubated with secondary antibodies in 1xPBST-2% normal serum for 2 h at RT. Following the counterstaining of DAPI (at 1ug/mL, cat#D3571, Thermo Fisher), sections were mounted on slides, allowed to dry, and cover slipped in fluoromount G (Southern Biotech). The following primary antibodies were used: Rabbit anti-ENO1 Antibody (at 1:100, Thermo Fisher, cat#PA5-21387 ), Goat anti-mouse Lipocalin-2 (LCN2) Antibody (at 1:50, R&D, cat#AF1857), Rat anti-GFAP (at 1:500, Thermo Fisher, cat# 13-0300, clone 2.2B10), anti-P2RY12 (at 1:100, Biolegend, cat#848002, clone S16007D), Rabbit anti-CLEC7A (at 1:200, Thermo Fisher, cat#PA-534382), Rabbit anti-Synapsin 1 (at 1:500 dilution, Synaptic Systems, cat#106 103), Guinea pig anti-PSD95 (at 1:250, Synaptic Systems, cat#124 014). The following secondary antibodies were used for staining the tissues: Goat anti-rabbit- Alexa Fluor Plus 647 (at 1:500, cat#A32733, Thermo Fisher), and goat anti-rat-Cy3 (at 1:500, cat#112-165-167, Jackson Immunoresearch), Goat anti-rat Cy5 (at 1:500, cat# 112-175-167, Jackson Immunoresearch), Goat anti-rabbit TRITC (at 1:500, cat# 111-025-144, Jackson Immunoresearch), Goat anti-guinea pig- DyLight™ 550 (at 1:500, cat# SA5-10095, Thermofisher), Donkey anti-goat Cy5 (at 1:500, Ab#6566, Abcam), Donkey Anti-rat-FITC (at 1:200, cat# *711-295-152*, Jackson Immunoresearch), Donkey anti-rabbit- Rhodamine Red (at 1:500, cat# 711-295-152, Jackson Immunoresearch).

#### Fluorescence microscopy and image analysis

Stained sections were examined and imaged using Olympus BX61 DSU fluorescence microscope with a Hamamatsu ORCA-FLASH4.0LT + SCMOS CAMERA. Image acquisition was targeted the CA1 for neuropathological analysis, while both the CA1 and dentate gyrus (DG) were imaged for ENO1 assessment in reactive astrocytes. Images were taken in stacks from three sections. All images were taken and processed using the integrated software program Olympus *cellSens* Software Version 4. Image analysis was performed using ImageJ (NIH) in a blinded fashion. For quantitative analysis, background fluorescence was subtracted for each image. The same auto-threshold method (Triangle, Renyientropy, or Otsu) was applied consistently across images within each analysis, and binary images were generated. Regions of interest (ROIs) were defined for each channel, and co- or tri-localized ROIs were created as appropriate. Co- or tri-localized signal areas were measured by applying these ROIs to the raw, unprocessed images. Area fraction was calculated as the percentage of the total imaged field of view occupied by co- or tri-localized signal. For visualization of representative images, brightness and contrast were adjusted uniformly across groups. While SYN1 and PSD95 reside in distinct compartments, their proximity within the limits of camera resolution at about 162nm (using x40 / 1.3 NA lens) makes them a preferred marker for detecting paired synapses.

### Statistical analyses in mice

Sample sizes were determined by previous publications^17,96,97^. Mice were randomly assigned to groups, and investigators were blinded to group allocation during data collection and analysis. Box plots were created in GraphPad Prism 10 (GraphPad Software), with center lines showing medians, boxes indicating the interquartile range, and whiskers extending from the minimum to the maximum values. Behavioral and histological data were analyzed in Prism 10. Two-sided Welch’s t-tests applied to rank-transformed data were used for all comparisons because Shapiro–Wilk tests indicated non-normality in several datasets and sample sizes were relatively small. For analyses involving three groups, the same Welch’s t-test approach was performed with Benjamini–Hochberg correction to account for multiple comparisons. Correlations were assessed by Spearman correlation analysis. For spatial acquisition (training), a two-way mixed-design ANOVA with Geisser-Greenhouse correction was employed, followed by Tukey’s multiple comparisons test.

## Supplementary Information


Supplementary Information.


## Data Availability

The data that support the findings of this study are available from the corresponding author, upon reasonable request.
