# Supplementary material for: Targeting hormone treatment for the brain in menopause
Source: Sci Rep. 2026 Sep 1;16:25901. doi: 10.1038/s41598-026-65603-4 (PMC13534712; doi:10.1038/s41598-026-65603-4)
Supplement: Supplementary file 1 [file 41598_2026_65603_MOESM1_ESM.pdf]

Voskuhl Menopause Brain Fog Assessment
Initial

How would you rate your difficulty with brain fog (as compared to 10 years ago)? *
- Severe difficulty (>50% worse)
- Moderate difficulty (25% worse)
- Mild difficulty (<10% worse)
- No difficulty

How would you rate your difficulty with quickly processing new information (as compared to 10 years ago)? *
- Severe difficulty (>50% worse)
- Moderate difficulty (25% worse)
- Mild difficulty (<10% worse)
- No difficulty

How would you rate your difficulty with remembering things (as compared to 10 years ago)? *
- Severe difficulty (>50% worse)
- Moderate difficulty (25% worse)
- Mild difficulty (<10% worse)
- No difficulty

How would you rate your difficulty with problem solving (as compared to 10 years ago)? *
- Severe difficulty (>50% worse)
- Moderate difficulty (25% worse)
- Mild difficulty (<10% worse)
- No difficulty

How would you rate your difficulty with concentrating (as compared to 10 years ago)? *
- Severe difficulty (>50% worse)
- Moderate difficulty (25% worse)
- Mild difficulty (<10% worse)
- No difficulty

How would you rate your difficulty with finding the right words during conversations (as compared to 10 years ago)? *
- Severe difficulty (>50% worse)
- Moderate difficulty (25% worse)
- Mild difficulty (<10% worse)
- No difficulty

Approximately what is your highest level of education (since this can affect brain fog)? *
- Less than 12 years
- 12 years (total years or high school grad or GED)
- 16 years (total years or college grad)
- More than 16 years (master's or other advanced degree or other total years of education)

How would you rate your overall quality of life now on a scale from 1-10? (10=great, 5=fair, 1=poor) *

VOSKUHL MENOPAUSE BRAIN FOG ASSESSMENT
12 MONTHS

Today, how would you rate your difficulty with brain fog? *
- Severe difficulty
- Moderate difficulty
- Mild difficulty
- No difficulty

Today, how would you rate your difficulty with concentrating? *
- Severe difficulty
- Moderate difficulty
- Mild difficulty
- No difficulty

Today, how would you rate your difficulty with remembering things? *
- Severe difficulty
- Moderate difficulty
- Mild difficulty
- No difficulty

Today, how would you rate your difficulty with quickly processing new information? *
- Severe difficulty
- Moderate difficulty
- Mild difficulty
- No difficulty

Today, how would you rate your difficulty with finding the right words during conversations? *
- Severe difficulty
- Moderate difficulty
- Mild difficulty
- No difficulty

Today, how would you rate your difficulty with problem solving? *
- Severe difficulty
- Moderate difficulty
- Mild difficulty
- No difficulty

Figure S1. The Voskuhl Menopause Brain Fog Assessment at Baseline (month 0, left) and Follow-up (month 12, right).

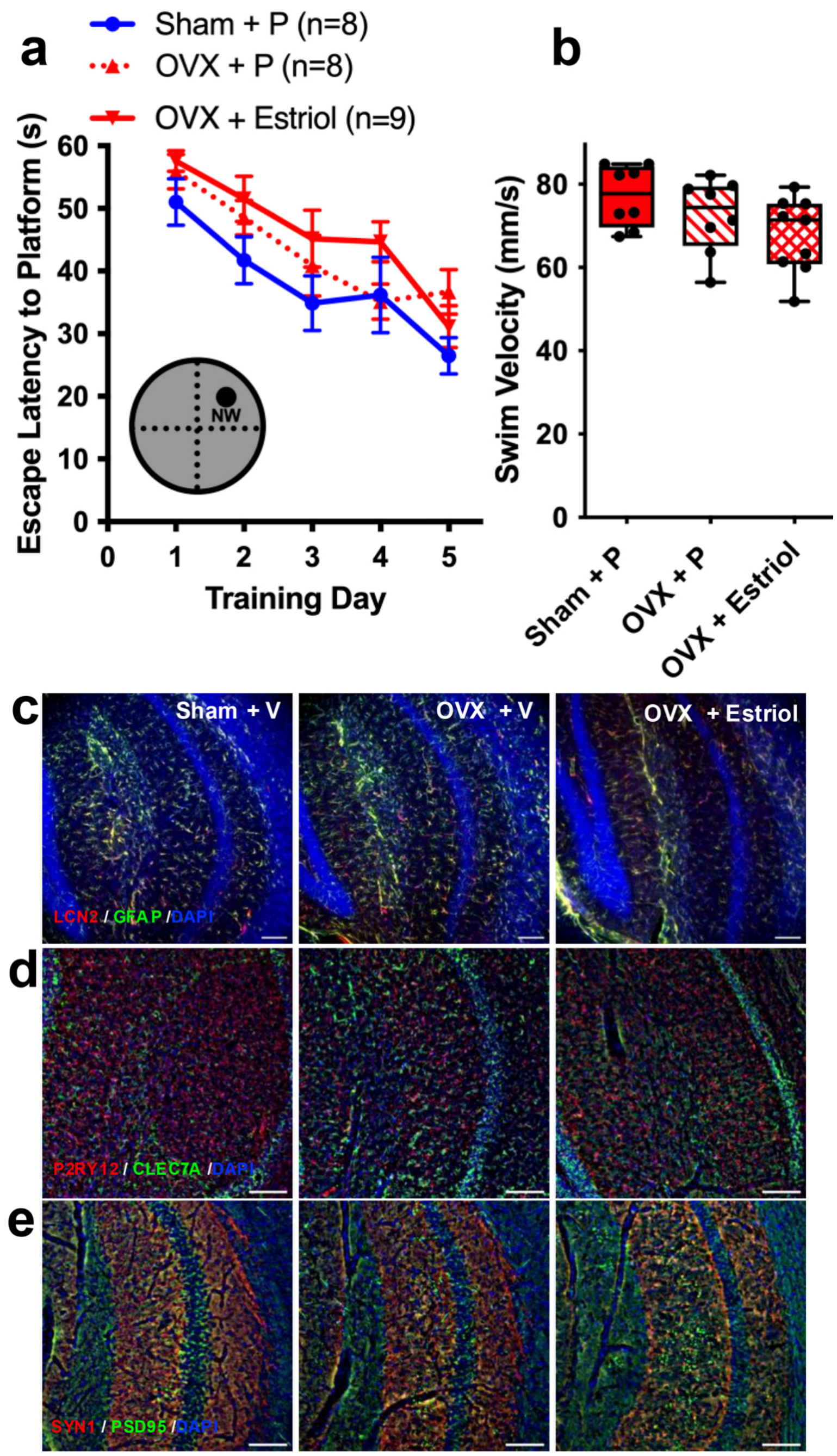

Figure S2. Female hormone deficit and estriol treatment at midlife did not affect spatial learning or motor function. (a) Spatial learning acquisition in the Morris Water Maze for Sham + V (solid blue), OVX + V (dotted red), and OVX + Estriol (solid red) midlife females. Data were analyzed using a two-way mixed-design ANOVA with Geisser-Greenhouse correction, followed by Tukey's multiple comparisons test. A significant main effect of Time ($p < 0.0001$), with all groups showing successful task acquisition (Day 1 vs. Day 5 latency: Sham + V: $p = 0.0101$; OVX + V: $p = 0.0040$; OVX + Estriol: $p = 0.0028$). No spatial learning acquisition differences among groups were

observed (Group effect: $p = 0.491$; Interaction: $p = 0.497$). Data are mean ± SEM. N=8-9. **(b)** Average swim speed on Day 5, confirming that ovariectomy and Estriol treatment did not affect locomotor activity ($p > 0.05$). N=8-9. All box plots with center lines showing the medians, boxes indicating the interquartile range, and whiskers indicating from the minimum and to the maximum values. Two-sided Welch's t-tests applied to rank-transformed data with Benjamini–Hochberg correction for multiple comparisons. **(c-d)** Representative 10× magnification images of (c) LCN2 (red), GFAP (green), merged images for colocalization (yellow) showing astrocyte reactivity, (d) CLEC7A (green), P2RY12 (red), merged image for colocalization (yellow) showing disease-associated microglia (DAM) , and (e) SYN1(red), PSD95 (green), merged images for colocalization (yellow) showing pre- and post-synaptic staining in the dorsal hippocampus, corresponding to Figure 3c-e. Nuclei were counterstained with DAPI (blue). Bar = 100um. Corresponding

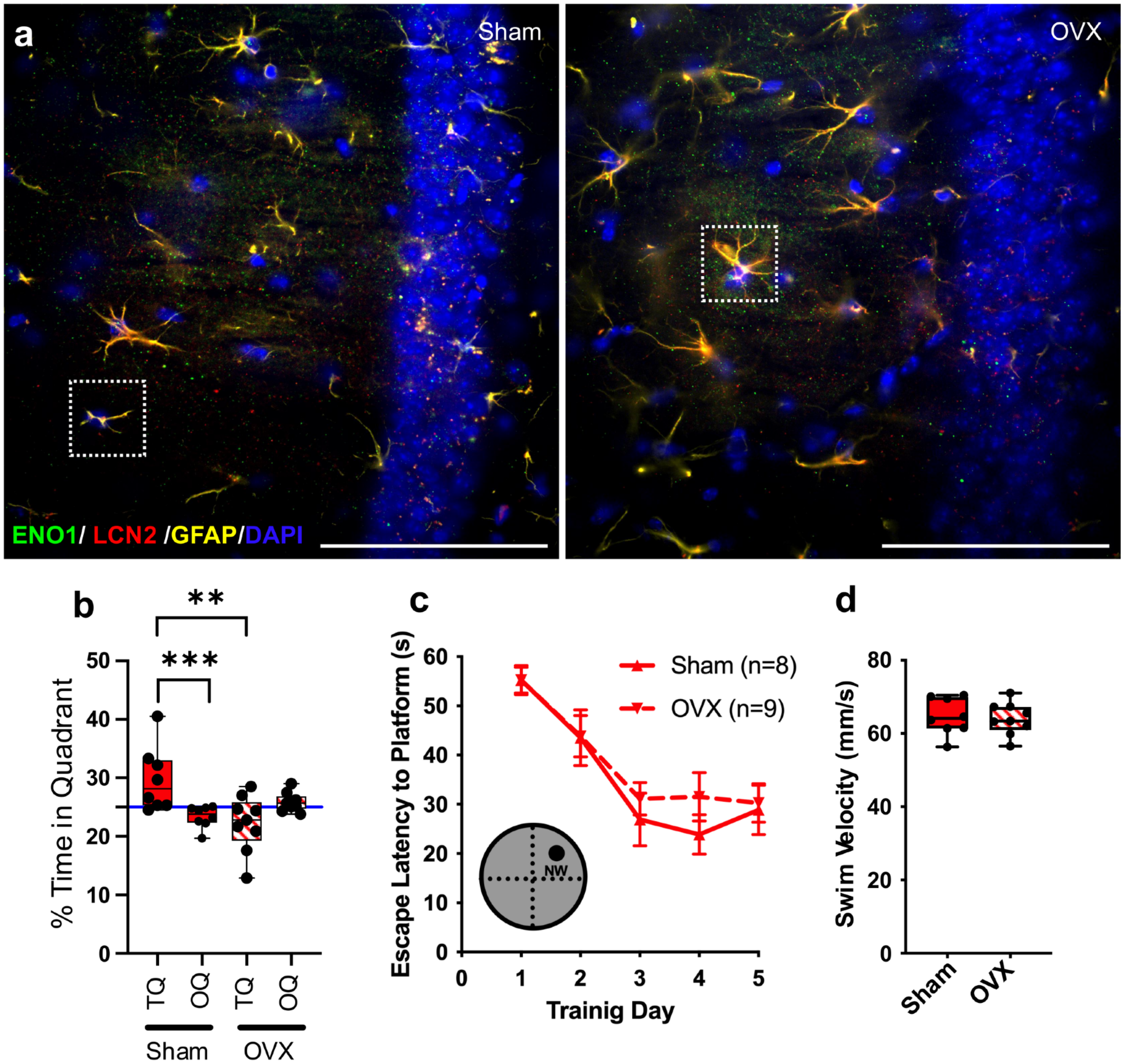

Figure S3. Female hormone deficit at midlife did not affect spatial learning acquisition or motor function. (a) Representative 40x magnification images of ENO1$^+$ (green), LCN2$^+$ (red), GFAP$^+$ (yellow), and merged images in the dorsal hippocampus, corresponding to Figure 4b. Dotted squares indicate the regions shown at higher magnification in the main figure. Nuclei stained with DAPI (blue). Bar=100um. **(b)** Probe trial performance 2 h post-training (platform removed). Target quadrant (TQ) preference was compared against the average of other quadrants (OQ) to validate spatial memory within each group. While Sham females showed a significant preference for the TQ ($p = 0.0009$), OVX females did not. (Note: Group comparison of % time in TQ is provided in Figure 4e). Mann–Whitney *U* test. N=8-9. **(c)** Spatial learning acquisition in the Morris Water Maze for Sham (solid red) and OVX (dotted red) midlife females. Both groups exhibited successful task acquisition ($p<0.0001$), with significant reductions in escape

latency between Day 1 and Day 5 (Sham: $p = 0.0073$; OVX: $p = 0.002$). No spatial learning acquisition differences among groups were observed (Group effect: $p = 0.51$; Interaction: $p = 0.759$). Data was analyzed via a two-way mixed-design ANOVA with Geisser-Greenhouse correction, followed by Tukey's multiple comparisons test. N=8-9. **(d)** Average swim speed on Day 5; no significant differences were observed, confirming that ovariectomy did not impair locomotor activity ($p > 0.05$). Two-sided Welch's t-tests applied to rank-transformed data. N=8-9. All box plots with center lines showing the medians, boxes indicating the interquartile range, and whiskers indicating from the minimum and to the maximum values.

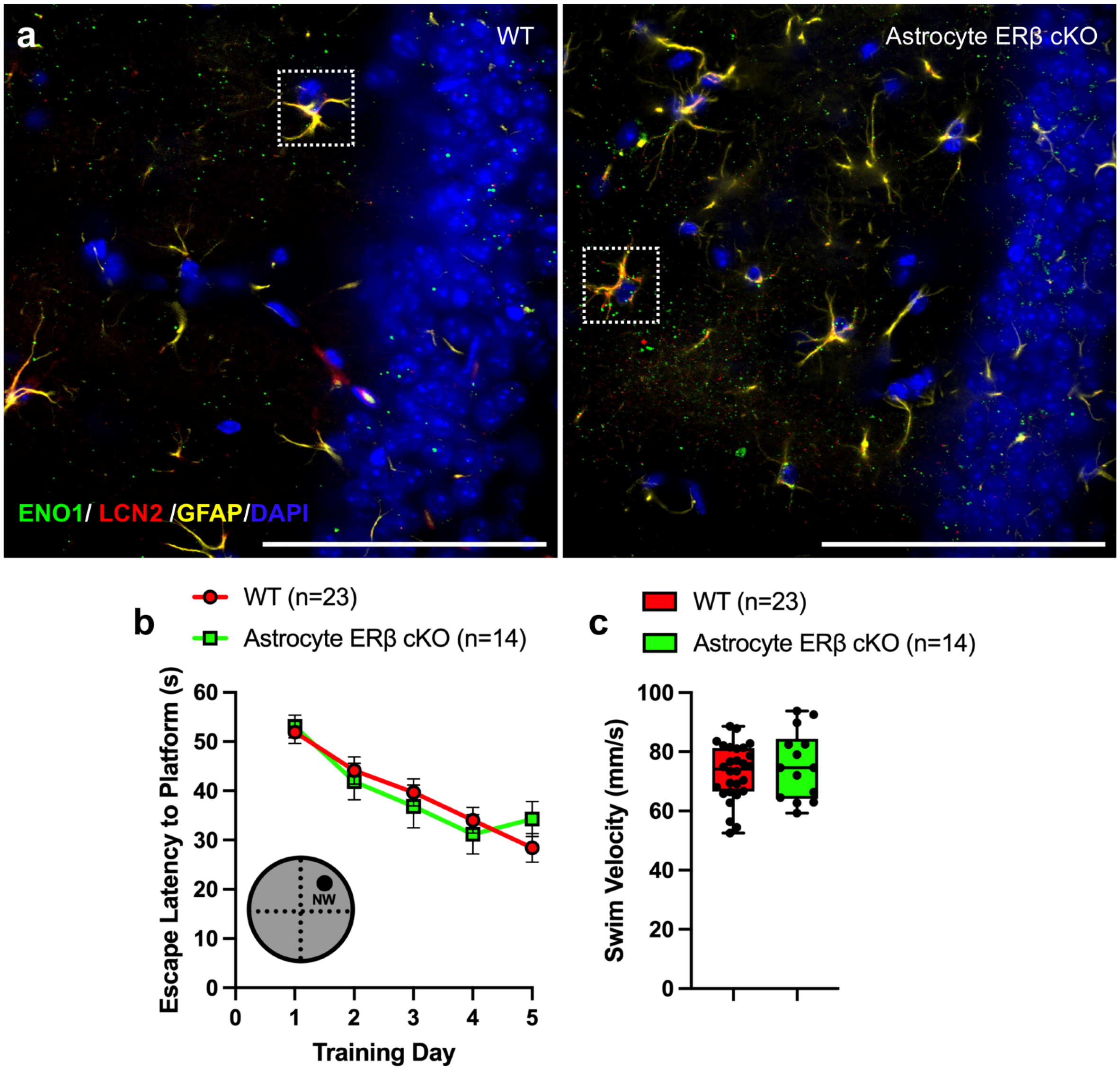

Figure S4. Selective deletion of ERβ in astrocyte did not affect spatial learning acquisition or motor function at midlife. (a) Representative 40x magnification images of ENO1$^+$ (green), LCN2$^+$ (red), GFAP$^+$ (yellow), and merged images in the dorsal hippocampus, corresponding to Figure 5b. Dotted squares indicate the regions shown at higher magnification in the main figure. Nuclei stained with DAPI (blue). Bar=100um. **(b)** Spatial learning acquisition in the Morris Water Maze for WT (red) and astrocyte ERβ cKO (green) midlife gonadally intact females. Both groups exhibited successful task acquisition ($p < 0.0001$), with significant reductions in escape latency between Day 1 and Day 5 (WT: $p < 0.0001$; astrocyte ERβ cKO: $p = 0.002$). No spatial learning acquisition differences among groups were observed (Group effect: $p = 0.9483$; Interaction: $p = 0.4305$). Data was analyzed via a two-way mixed-design ANOVA with Geisser-Greenhouse correction, followed by Tukey's multiple comparisons test. N=14-

23. **(c)** Average swim speed on Day 5; no significant differences were observed, confirming that ovariectomy did not impair locomotor activity ($p > 0.05$). Two-sided Welch's t-tests applied to rank-transformed data. N=14-23. All box plots with center lines showing the medians, boxes indicating the interquartile range, and whiskers indicating from the minimum and to the maximum values.

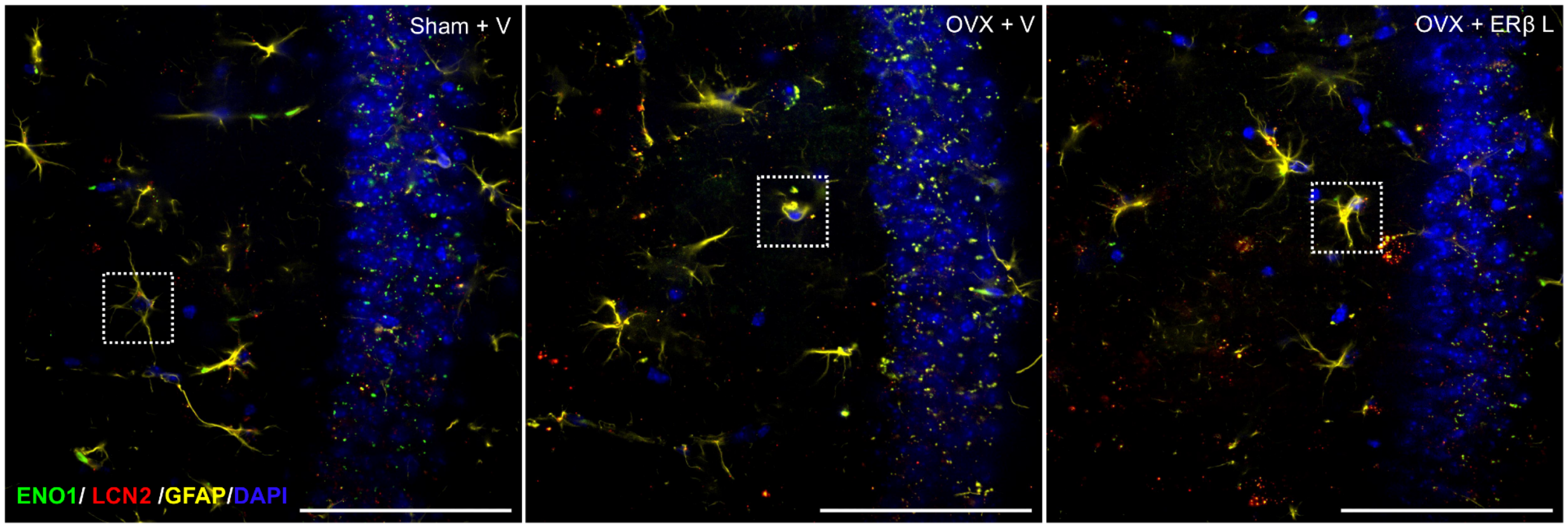

Figure S5: Representative 40x magnification images of ENO1⁺ (green), LCN2⁺ (red), GFAP⁺ (yellow), and merged images in the dorsal hippocampus, corresponding to Figure 6b. Dotted squares indicate the regions shown at higher magnification in the main figure. Nuclei stained with DAPI (blue). Bar=100um.

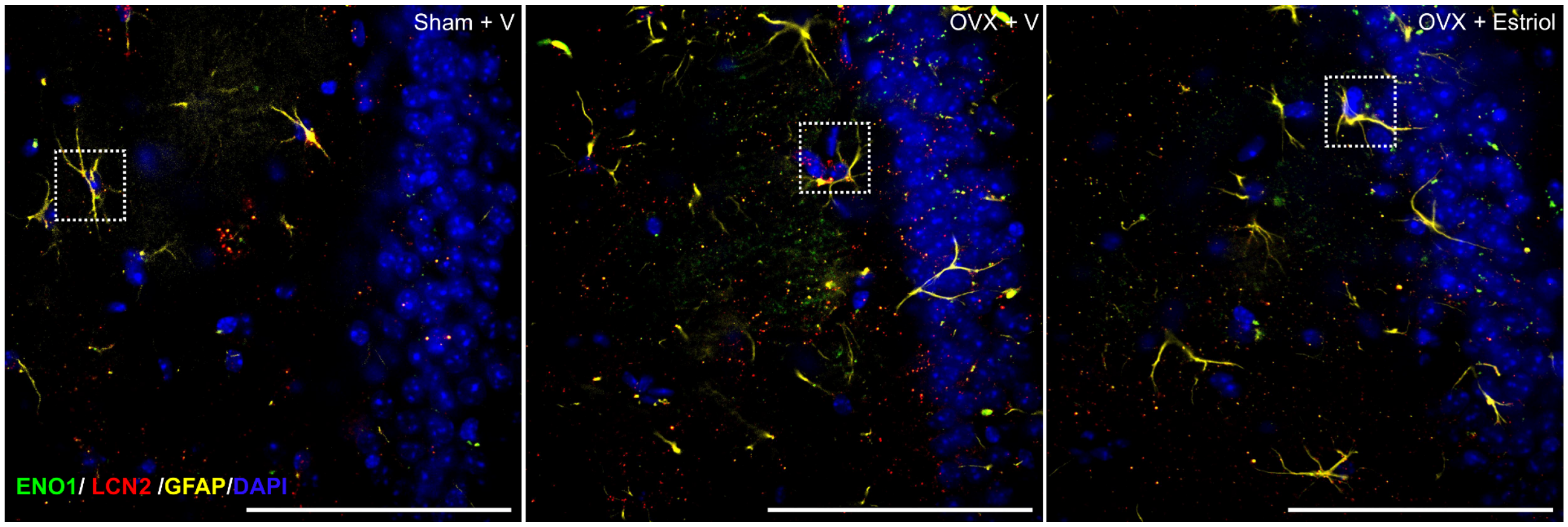

Figure S6: Representative 40x magnification images of ENO1⁺ (green), LCN2⁺ (red), GFAP⁺ (yellow), and merged images in the dorsal hippocampus, corresponding to Figure 7a. Dotted squares indicate the regions shown at higher magnification in the main figure. Nuclei stained with DAPI (blue). Bar=100um.

Table S1: Summary of Animal Allocation, Exclusions, and Final Sample Sizes

Experiment	***Estriol Treatment (Fig. 3, 7)***	***Menopause Model (Fig 4)***	***Astrocyte ERβ cKO (Fig 5)***	***ERβ Ligand (Fig 6)***
Group	Sham + V / OVX + V / OVX + Estriol	Sham + V / OVX + V	WT / Astrocyte ERβ cKO	Sham + V / OVX + V / OVX + ERβ L
Initial N	42 (16/16/10)<br>* 2 cohorts	21 (10/11)	44 (26/18)<br>* 2 cohorts	46 (16/16/14)<br>* 2 cohorts
Post-Surgery N	N=41 (16/15/10)	N=21 (10/11)	No Surgery	N=45 (16/15/14)
Health Exclusions	N=1 (1/0/0)	N=2 (1/1)	N=3 (1/2)	N=2 (1/0/1)
Behavioral Exclusions (MWM) - Swim issue	N=3 (1/1/1)<br>*1 cohort	N=2 (1/1)	N=4 (2/2)	N/A
Final N Analyzed (Behavior)	N=25 (8/8/9)<br>*1cohort	N=17 (8/9)	N=37(23/14)<br>* 2 cohorts	N/A
Final N Analyzed (Neuropathology)	N=24 -25<br>(8/7/9 or 10#)<br>*1 cohort	N/A	N/A	N/A
Final N Analyzed (ENO1 Protein Expression)	N=40 (15/15/10)<br>* 2 cohorts	N=17 (8/9)	N=15 (7/8)<br>*1 cohort	N=42 (15/ 14 or 15#/ 12 or 13#)
Reason for N Variation	MWM in 1 cohort; ENO1 in 2 cohorts.<br># tissue damage.	Same mice for both assays.	ENO1 analysis from 1 cohort only.	MWM previously reported [Itoh et al Nat. Comm.].<br># tissue damage.
